# Long-Term Changes in the Distribution and Abundance of Nine Deep-Water Skates (Arhynchobatidae: Rajiformes: Chondrichthyes) in the Northwestern Pacific

**DOI:** 10.3390/ani12243485

**Published:** 2022-12-09

**Authors:** Alexei M. Orlov, Igor V. Volvenko

**Affiliations:** 1 Laboratory of Oceanic Ichthyofauna, Shirshov Institute of Oceanology, Russian Academy of Sciences, 117218 Moscow, Russia; 2 Laboratory of Behavior of Lower Vertebrates, A.N. Severtsov Institute of Ecology and Evolution, Russian Academy of Sciences, 119071 Moscow, Russia; 3 Department of Ichthyology and Hydrobiology, Tomsk State University, 634050 Tomsk, Russia; 4 Department of Ichthyology, Dagestan State University, 367000 Makhachkala, Russia; 5 Laboratory of Marine Biology, Caspian Institute of Biological Resources, Dagestan Federal Research Center, Russian Academy of Sciences, 367000 Makhachkala, Russia; 6 Laboratory for Modeling of Biological Processes, Pacific Branch of the Russian Federal Research Institute of Fisheries and Oceanography, 690091 Vladivostok, Russia

**Keywords:** spatial distribution, catch dynamics, latitudinal range, western Bering Sea, Sea of Okhotsk, northwestern Sea of Japan, Sakhalin Island, Pacific waters, Kuril Islands, Kamchatka

## Abstract

**Simple Summary:**

Changes in the spatial distribution and abundance of common skate species of the genus *Bathyraja* in the Russian waters of the Northwestern Pacific, where their fishery is considered promising, but still not developed, are traced. Over the past six decades, the boundaries of their ranges have slowly fluctuated near some average annual position and abundance trends have coincided with previously identified ecosystem rearrangements under the influence of climatic and oceanological changes. It will be useful to compare the results obtained with abundance trends in the near future, when fishing for these species is expected to intensify here, as well as with other areas where fishing pressure for these species already exists.

**Abstract:**

Based on the analysis of long-term data from bottom trawl surveys (1977–2021), changes in the spatial distribution, position of the boundaries of the ranges and the catch rates of the nine most common deep-sea skates of the genus *Bathyraja* in the Russian waters of the Northwestern Pacific (*B. violacea*, *B. aleutica*, *B. matsubarai*, *B. maculata*, *B. bergi*, *B. taranetzi*, *B. minispinosa*, *B. interrupta,* and *B. isotrachys*) are considered. During the surveyed period, significant changes in the spatial distribution were observed, which are probably due to both subjective reasons (changes in the number of trawling stations, surveyed depths, etc.) and climatic changes. No monotonous displacement of the northern or southern boundaries of the range or its center in one direction was observed in any area of any species during the entire observation period. At the same time, shifts in the boundaries of the ranges of different species in different areas occurred for different decades, i.e., the boundaries of the ranges slowly fluctuated or “pulsed” near some average annual position. In general, from the 1970s to the 1980s, the number of skates grew; from the 1980s to the 1990s, it decreased; from the 1990s to the 2000s, it fluctuated at the achieved level; from the 2000s to the 2010s, it grew again; and from the 2010s to the 2020s, it decreased again. These trends coincide with previously identified ecosystem rearrangements under the influence of climatic and oceanological changes. The identification of links between changes in spatial distribution, range boundaries and catch rates with climatic and oceanological factors require separate additional studies.

## 1. Introduction

Representatives of the order Rajiformes are widely distributed in the North Pacific Ocean. They play an important role in marine ecosystems [1], being mainly top predators consuming many valuable commercial invertebrates and fish [2,3,4,5,6,7,8,9].

Skates have biological features common to most cartilaginous fish (slow growth rates, late sexual maturation and low reproductive rates), which makes their stocks extremely vulnerable to fishing [10]. However, they are commercially important fishing targets in many countries [11], particularly in Southeast Asia, where skate wings are used for consumption [12]. The flesh of skates is rich in nutrients and contains an almost complete set of essential amino acids. Their livers, being rich in vitamin A, though less so than those of sharks, can be used for the extraction of oil for veterinary and medical purposes. Skates have strong and thick skin that may be used for the manufacture of many leather articles. The flesh of skates is suitable for processing to fish meat jelly, which is used in the preparation of various Japanese national dishes [12,13,14,15].

The biomass of skates within Russian waters of the northwestern Pacific amounted to 677 thousand tons [16], and in some areas they account for up to 10% of the total biomass of groundfish [17]. Their fishing in Russian waters has long been considered promising [18,19], but has not yet been developed. In the Far Eastern Fisheries basin of Russia in the 1990s, only 11.4–11.7 thousand tons were recommended for harvesting; and in the 2000s, 7.2–11.9; in the 2010s, 11.2–14.0; and in the 2020s, 11.2–11.3 thousand tons (first within estimated total allowable catch—TAC, then within possible catch—PC or recommended catch—RC). Skates were not separated by species, but their actual total catch never reached the recommended values, since these fish got on board Russian fishing vessels as bycatch only, were not retained and utilized and were subsequently discarded [20,21,22]. The situation has changed in recent years after some Russian companies started to export skate wings to China and the British Virgin Islands at prices between 0.9 and 1.3 USD per kg, while they also may be profitably marketed in Japan and Korea [23,24].

According to long-term monitoring data [25,26], at least 28 species of skates are regularly found within the Russian waters and adjacent areas of the North Pacific, and all of them have commercial value [27,28]. To date, there is published information about the general features of the spatial distribution and dynamics of catches of the most common and some rare species in the Pacific waters off the North Kuril Islands and southeastern Kamchatka [17,29,30,31]. Recently, data on the spatial distribution and dynamics of catches of Alaska skate *Bathyraja parmifera*, Okhotsk skate *Bathyraja violacea* and Aleutian skate *Bathyraja aleutica* throughout the North Pacific have also been described [32,33,34]. Additionally, there is information on the distribution and biology of the bottom skate *Bathyraja bergi* in the Russian waters of the Sea of Japan [35]. Nevertheless, the features of spatial distribution, dynamics of skate catches and their long-term changes have not been considered in detail for Russian waters of the northwestern Pacific. Meanwhile, insufficient information prevents the evaluation of correct values of the TAC, PC or RC differentiated by species and area, and the subsequent rational exploitation of skates stocks in the Russian waters of the northwestern Pacific, which during the period of increasing intensity of their fishing may negatively affect the condition of populations of these fish [36].

The goal of this paper is to identify changes in the spatial distribution and catch rates of the nine most common skates within the Russian Exclusive Economic Zone (EEZ) of the Northwestern Pacific (the northwestern Sea of Japan and the Sea of Okhotsk, western Bering Sea and Pacific waters off the Kuril Islands and Eastern Kamchatka) deep-water skates of the genus *Batyraja* based on the analysis of long-term data of bottom trawl surveys.

## 2. Material and Methods

**Figure 1:**
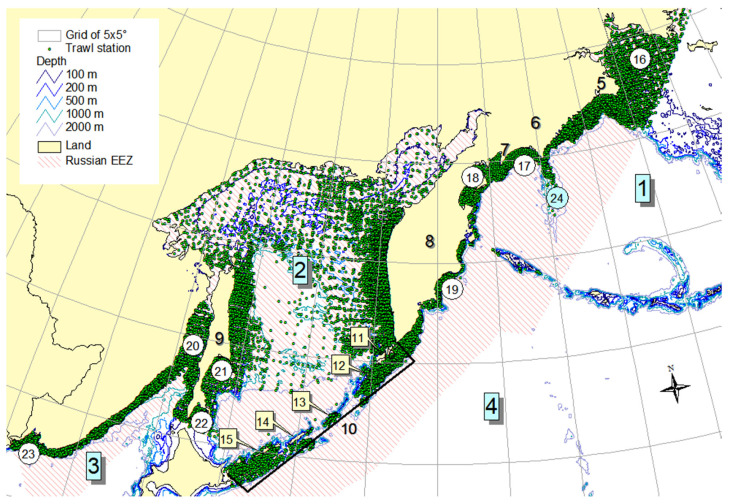
Location of trawl stations in the surveyed area. The numbers indicate the geographical names mentioned in the text: 1 — Bering Sea, 2 — Sea of Okhotsk, 3 — Sea of Japan, 4 — Pacific Ocean, 5 — Navarin Cape, 6 — Koryak coast, 7 — Goven Cape, 8 — Kamchatka, 9 — Sakhalin, 10 — Kuril Islands, 11 — Paramushir Isl., 12 — Onekotan Isl., 13 — Simushir Isl., 14 — Urup Isl., 15 — Iturup Isl., 16 — Gulf of Anadyr, 17 — Olyutor Bay, 18 — Karagin Bay, 19 — Kronotsk Bay, 20 — Tatar Strait, 21 — Terpeniya Bay, 22 — Aniva Bay, 23 — Peter the Great Bay, 24 — Shirshov Underwater Ridge.

Material was obtained from database [37] supplemented with data from the recent bottom trawl surveys conducted by the Pacific Branch of the Russian Federal Research Institute of Fisheries and Oceanography through 2021. We selected from it trawl stations located within the Russian EEZ (there were 39,420 of them, see Figure 1) and calculated the occurrence of all skate species over the research period (1977–2021). Species encountered less than 100 times are not included in the list. *B. parmifera* and *Bathyraja smirnovi* were also excluded from it, since until recently, information about the distribution of these two morphologically similar species was very contradictory [27,32,38,39,40,41,42,43,44]; as a result, research teams confused them with each other. Thus, nine of the most common species were identified, which became the objects of this study (Table 1).

Identification of skates to species level during past surveys by research teams was conducted using available guides [45,46,47]. During recent surveys, scientists for this purpose additionally used publications [48,49]. Scientific (Latin) and common names of skates used in this paper are provided according to the most authoritative sources [50,51]. 

To analyze long-term changes in their distribution and abundance, we divided the time scale into six decades: (1) 1970s, (2) 1980s, (3) 1990s, (4) 2000s, (5) 2010s and (6) 2020s. The sample sizes thus obtained are shown in Table 2.

For each species in each decade and area, we found the maximum (max), minimum (min) and average (avg) latitudes of all catches to determine the northern and southern boundaries of the range, and its center in decimal degrees. The abundance of the species at each capture point, as well as the average for decades and areas, was estimated by catch per unit of effort (CPUE). For unit of effort, we took the area of a trawl haul (in square kilometers), which was calculated by multiplying the trawl horizontal opening by hauling distance, i.e., the unit of measure of CPUE is ind./km^2^.

In addition to the decadal changes in the four variables, which are described in detail in this paper, we estimated their total trends for all time using four simple regression models: (1) Linear: *y = a + b · x*, (2) Logarithmic: *y = a + b · ln(x)*, (3) Exponential: *y = exp(a + b · x)* and (4) Multiplicative: *y = a x^b^*, where *x* is the decade number and *y* is the variable being approximated. In each particular case the best model was chosen by the minimum of the residual and *p*-value, and the maximum of the correlation coefficient (*r*) modulo. We hoped that the *r* sign would help classify trends as positive or negative. However, the variations in the variables turned out to be not monotonous, poorly approximated by smooth lines. Accordingly, the *r* values in most cases turned out to be non-significant at the 95% confidence level. All graphs, their equations, and *r* and *p* values are given in the Supplement, and the results are shown in the last columns of Table 3 and Table 4.

## 3. Results

### 3.1. Okhotsk Skate

**Figure 2:**
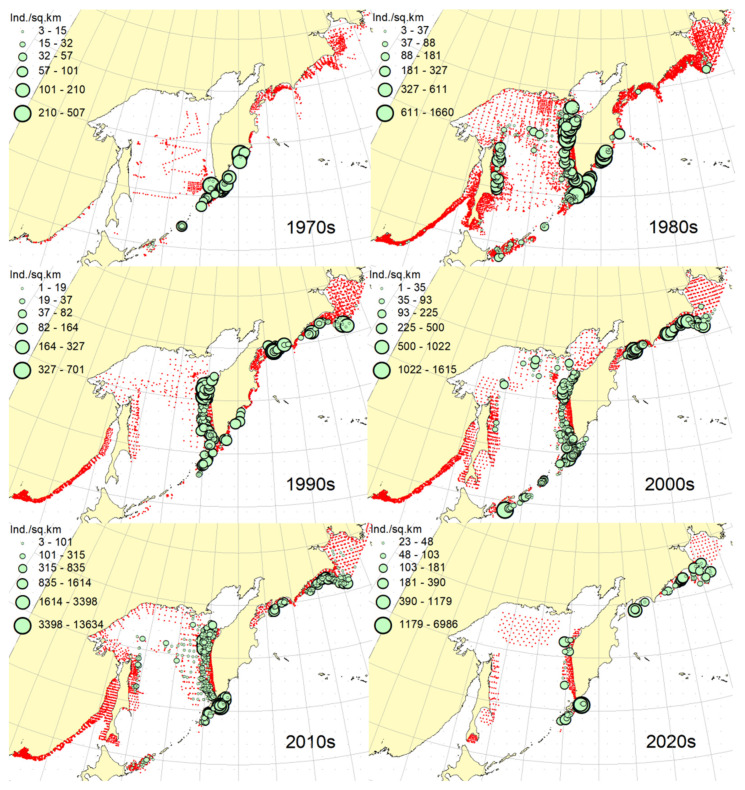
Decadal changes in the spatial distribution of the Okhotsk skate *Bathyraja violacea* in the Northwestern Pacific. Here and on Figure 2, Figure 3, Figure 4, Figure 5, Figure 6, Figure 7, Figure 8 and Figure 9, red dots indicate stations where this species was not recorded.

***Spatial distribution*** (Figure 2). During the research period, the spatial distribution of the Okhotsk skate has changed significantly. In the first decade, it was caught only in the waters off the central and northern Kuril Islands and southern Kamchatka. The maximum catches were taken off the southwestern Kamchatka in the Sea of Okhotsk and off the central part of the East Kamchatka coast in the Pacific Ocean. In the 1980s, this species was found most widely within the surveyed area, i.e., from the Southern Kuril Islands in the south to Navarin Cape in the western Bering Sea in the north. In the Sea of Okhotsk, it was observed almost everywhere except in the southern part of the sea, and the maximum catches were recorded off both coasts of Kamchatka and the northern Kuril Islands. In the next decade, the distribution area of the Okhotsk skate was reduced. It was recorded only in the western Bering Sea, off the northern Kuril Islands, western and southeastern Kamchatka. Maximum catches were recorded in the Sea of Okhotsk off the central part of the West Kamchatka coast. In the 2000s, the distribution area of the Okhotsk skate expanded again. It began to occur off the eastern Sakhalin, in the northern Sea of Okhotsk, and off the southern and central Kuril Islands, but ceased to occur near the East Kamchatka coast. The maximum catches during this period were recorded off the southern and northern Kuril Islands and in the western Bering Sea. The spatial distribution of the Okhotsk skate in the 2010s did not fundamentally differ from that in the 2000s. However, the maximum catches shifted to the waters of the western and southeastern coasts of Kamchatka, and also remained off the northern Kuril Islands. At the beginning of the current decade, the Okhotsk skate disappeared from catches in the northern Sea of Okhotsk and off the eastern Sakhalin, but it was still observed in the western Bering Sea, off the northern Kuril Islands and western Kamchatka with maximum catches in the waters off the northern Kuril Island.

**Figure 3:**
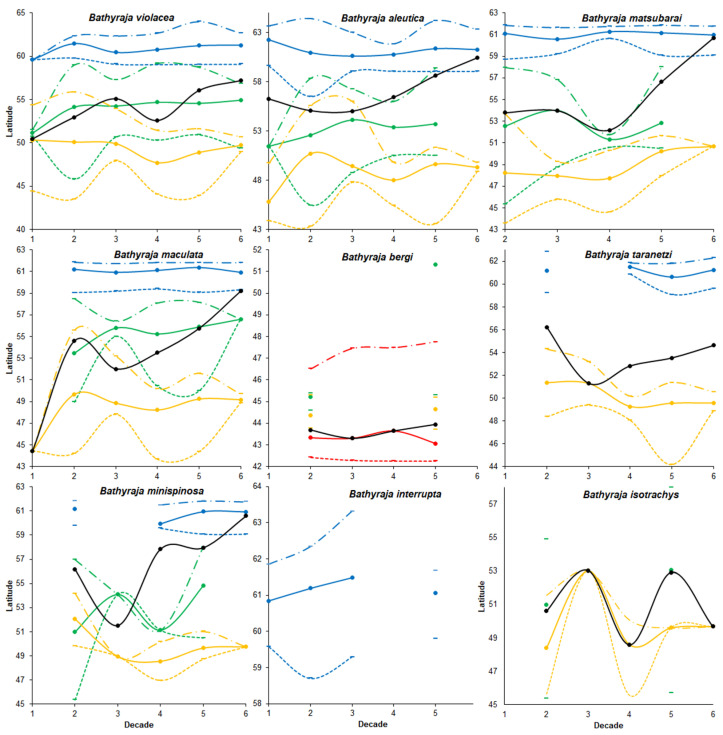
Decadal changes in the position of the range of nine skate species in the Northwestern Pacific. The Bering Sea is in blue, Sea of Okhotsk in green, Sea of Japan in red, and Pacific Ocean in yellow, and all areas combined are in black. The northern boundaries of the ranges are shown by dash-dotted lines, southern boundaries by dashed lines, and middle of the ranges by solid lines with circles.

***Boundaries of the ranges*** (Figure 3, Supplementary Figures S1–S3). In general, the distribution center of the Okhotsk skate shifted significantly to the north during the research period. At the same time, the trends in different areas differed significantly. Whereas the northern distribution boundaries in the western Bering Sea and Sea of Okhotsk have shifted northward, in the Pacific waters of the Kuril Islands and eastern Kamchatka, they shifted south. Similar trends in orientation, but not so pronounced, were noted in relation to distribution centers. The southern borders of the ranges showed slightly different dynamics. Whereas in the Sea of Okhotsk and Pacific waters the borders shifted northward, in the Bering Sea, they showed a weak but opposite trend.

**Figure 4:**
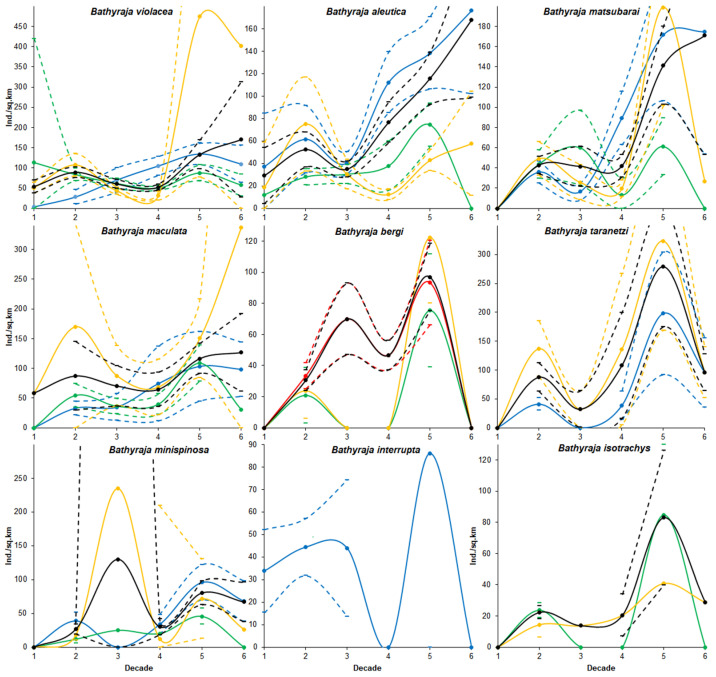
Decadal changes in the CPUE of nine skate species in the Northwestern Pacific. The Bering Sea is in blue, Sea of Okhotsk in green, Sea of Japan in red, and Pacific Ocean in yellow, and all areas combined in black. The solid lines with circles indicate the average CPUEs, and the dotted lines indicate the 95% confidence intervals.

***Catch dynamics*** (Figure 4 and Supplementary Figure S28). In general, during the research period, the value of catches per unit effort of Okhotsk skate increased by almost 3 times. At the same time, an increase in catches was noted in the western Bering Sea and Pacific waters (especially sharp, starting from the fourth decade). In the Sea of Okhotsk, on the contrary, a decrease in the catches rates (especially at the beginning of the research period) was observed.

### 3.2. Aleutian Skate

**Figure 5:**
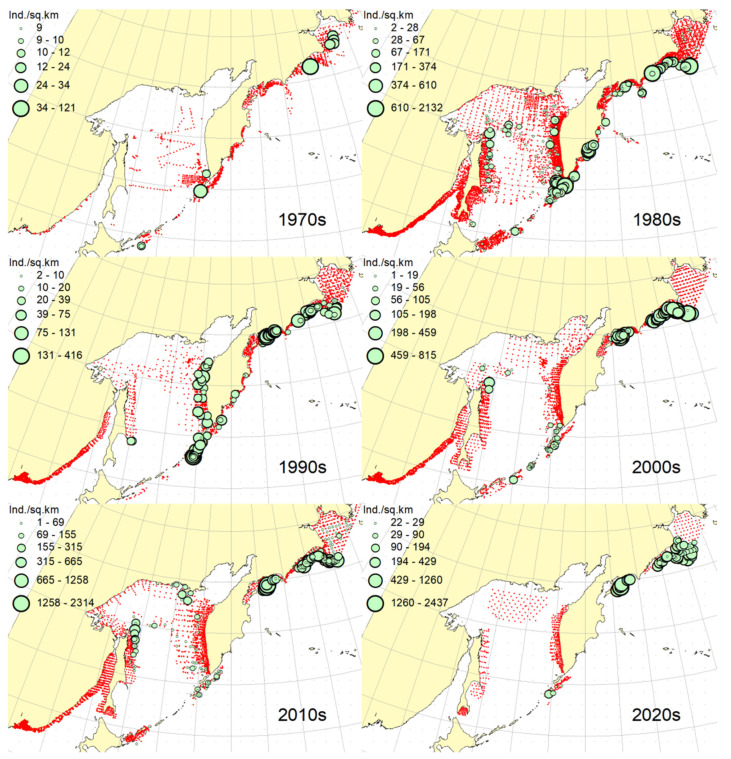
Decadal changes in the spatial distribution of the Aleutian skate *Bathyraja aleutica* in the Northwestern Pacific. Designations as in Figure 1.

***Spatial distribution*** (Figure 5). In the 1970s, the Aleutian skate was sporadically observed in catches off the southern and northern Kuril Islands, off southwestern Kamchatka and in the western Bering Sea. Its most frequent captures were recorded in the Gulf of Anadyr. The maximum catches were recorded in the central part of the Koryak coast and in the Pacific waters of the northern Kuril Islands. During the next decade, this species was most widely distributed within the study area. Its captures were recorded from the southern Kuril Islands in the south to the northern part of the Gulf of Anadyr in the north. In the Sea of Okhotsk, it was found almost everywhere except in the southwestern part of the sea. The maximum catches were in the Pacific waters of the northern Kuril Islands, off the central parts of the East Kamchatka and Koryak coasts and off the Navarin Cape. In the third decade, catches of the Aleutian skate were recorded mainly along the western and eastern coasts of Kamchatka, in the Pacific waters of the northern Kuril Islands and in the western Bering Sea. Several catches were recorded off the Eastern Sakhalin to the east of Terpeniya Bay. The maximum catches were recorded in the Olyutor Bay of the Bering Sea and in the Pacific waters of the northern Kuril Islands. In the 2000s, the distribution of the Aleutian skate changed significantly. Catches from Eastern Sakhalin have shifted far to the north. Off the western Kamchatka and northern Kuril Islands, their values have significantly decreased, and the captures themselves have shifted south to the Simushir Island. In the western Bering Sea, the distribution pattern has practically not changed, but the value of catches has increased significantly. In the next decade, the distribution of this species changed again. The occurrence has increased near northeastern Sakhalin, in the northern Sea of Okhotsk and off western Kamchatka. Individual catches have been recorded in the Pacific waters of the southern Kuril Islands. In the western Bering Sea, the catches in the Gulf of Anadyr have not only increased, but they have also been recorded almost within the entire Gulf of Anadyr. For several decades, the maximum catches were recorded in the Olyutor Bay, in the central part of the Koryak coast and off the Navarin Cape. At the beginning of the current decade, the Aleutian skate was found mainly in the western Bering Sea and also in a few catches in the Pacific waters of the northern Kuril Islands.

***Boundaries of the ranges*** (Figure 3, Supplementary Figures S4–S6). In general, the research area was characterized by a shift of the distribution center in the north direction. At the same time, the northern border of the range has shifted significantly to the north in the Sea of Okhotsk. In the Bering Sea and Pacific waters, on the contrary, it has shifted to the south (in the latter area it is noticeably stronger). The center of the range has shifted to the north in the Sea of Okhotsk and Pacific waters and to a lesser extent to the south in the Bering Sea. The southern distribution boundaries in all three areas shifted to the north (most noticeably in the last two areas).

***Catch dynamics*** (Figure 4, Supplementary Figure S29). During the research period, the value of the average catch per unit effort for all areas increased several times. At the same time, catches of the Aleutian skate in the western Bering Sea demonstrated the maximum growth. To a lesser extent, the increase in catches was typical for Pacific waters. In the Sea of Okhotsk, despite a sharp decrease in the number of catches per unit effort in recent years, the general trend showed a certain increase in them as a whole for the entire period of research.

### 3.3. Dusky-Purple Skate

**Figure 6:**
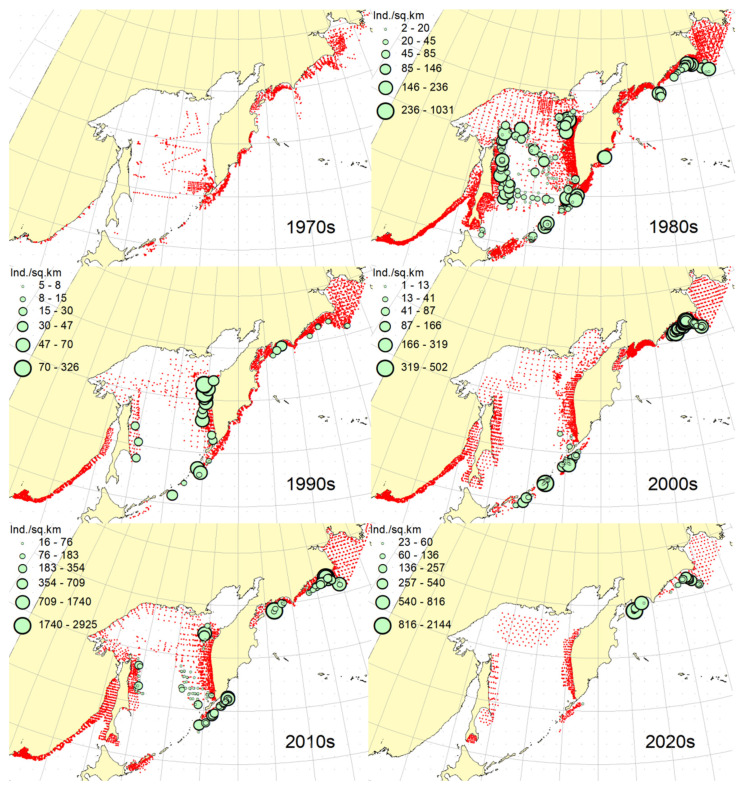
Decadal changes in the spatial distribution of the dusky-purple skate *Bathyraja matsubarai* in the Northwestern Pacific. Designations as in Figure 1.

***Spatial distribution*** (Figure 6). In the first decade, there were no captures of the dusky-purple skate within the surveyed area. In the 1980s, it was most widely distributed, occurring from the southern Kuril Islands in the south to m. Navarin in the Bering Sea in the north. In the Sea of Okhotsk, it was found almost everywhere, with the exception of a deep-water basin in the southwestern part of the sea. The maximum catches in this period were typical for the waters of Eastern Sakhalin, central and northern Kuril Islands, eastern Kamchatka and the western Bering Sea. In the 1990s, the area of occurrence and number of catches of this species decreased significantly. The main catches during this period were observed in the waters of Western Kamchatka. The second most common area of occurrence was the western Bering Sea. Occasional catches were also recorded off Eastern Sakhalin and in the Pacific waters of the central Kuril Islands. In the next decade, the range of the dusky-purple skate decreased even more. It has practically disappeared from catches in the waters of Western Kamchatka and Eastern Sakhalin but has become more numerous in the western Bering Sea and Pacific waters of the Kuril Islands from Paramushir Island to Iturup Island. In the 2010s, the area of distribution of this species increased markedly. It reappeared in the waters of Eastern Sakhalin and Western Kamchatka. In the Pacific waters of the Kuril Islands, catches shifted northward; in the western Bering Sea, the occurrence expanded westward to the Karagin Bay. The maximum catches were recorded in the western Bering Sea. In the early years of the current decade, the dusky-purple skates were recorded in the western Bering Sea only and also caught once in the Pacific waters of southeastern Kamchatka. The maximum catches were recorded in the Olyutor Bay of the Bering Sea.

***Boundaries of the ranges*** (Figure 3, Supplementary Figures S7–S9). In general, the period of research was characterized by a significant shift of the distribution center to the north. At the same time, the position of the northern border of the range in the Bering Sea has not changed, while in the Sea of Okhotsk and Pacific waters it has shifted to the south. The center of distribution in the Bering Sea also did not undergo displacement; while in the Sea of Okhotsk, it shifted somewhat southward; and in Pacific waters, on the contrary, to the north. The southern boundary of the range in all three areas shifted northward (to a lesser extent in the western Bering Sea).

***Catch dynamics*** (Figure 4, Supplementary Figure S30). In general, for the research period, catches per unit effort increased many times over the course of six decades. At the same time, the maximum increase in catches was recorded in the Pacific waters and western Bering Sea. In the former area, after a sharp increase in the catch rates in the fifth decade, an equally sharp decline has been noted in recent years. In the Sea of Okhotsk during the research periods, there was an alternation of ups and downs of the catch rates, as a result of which the overall trend turned out to be weakly positive.

### 3.4. Whiteblotched Skate

**Figure 7:**
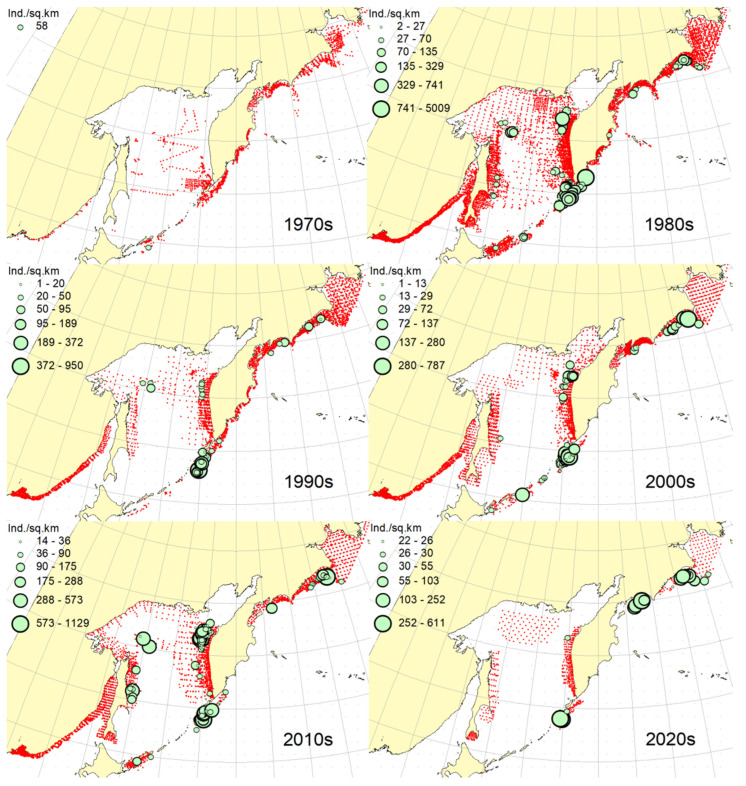
Decadal changes in the spatial distribution of the whiteblotched skate *Bathyraja maculata* in the Northwestern Pacific. Designations as in Figure 1.

***Spatial distribution*** (Figure 7). In the 1970s, the whiteblotched skate in the research area was marked by a single capture in the waters of the southern Kuril Islands to the east of the Iturup Island. In the second decade, it was found in catches from southern Kuril Islands in the south to Navarin Cape in the Bering Sea in the north. In the Sea of Okhotsk, it was recorded near Eastern Sakhalin; in the northwestern part of the sea, off Western Kamchatka; and the northern Kuril Islands. The maximum catches were typical for the waters of the northern Kuril Islands and southeastern Kamchatka. In the 1990s, the range of the whiteblotched skate significantly decreased. It disappeared from catches in the waters of Eastern Sakhalin, southwestern Kamchatka and the Sea of Okhotsk waters off the northern Kuril Islands. The main area of occurrence and maximum catches remained the Pacific waters of the northern Kuril Islands. The second most important area was the western Bering Sea. The pattern of catch distribution of this species has not fundamentally changed in the next decade, except for individual catches off Eastern Sakhalin and in the Pacific waters of the southern and central Kuril Islands. The main area of occurrence and maximum catches were still the Pacific waters of the northern Kuril Islands. The value of catches in the waters of Western Kamchatka and the western Bering Sea has increased markedly in comparison with the previous decade. A similar distribution pattern of whiteblotched skate catches was typical for the 2010s. The only difference between these two adjacent decades was the appearance of this species in catches off Eastern Sakhalin and the northwestern Sea of Okhotsk. In addition to the Pacific waters of the northern Kuril Islands and the western Bering Sea, the maximum catches were also recorded in the waters of Western Kamchatka. In the current decade, the western Bering Sea has remained the main area of occurrence and maximum catches of whiteblotched skate. It was also recorded by single capture off the northwestern Kamchatka. Catches in the Pacific waters of the northern Kuril Islands have become occasional, although they have retained their high value.

***Boundaries of the ranges*** (Figure 3, Supplementary Figures S10–S12). In general, in the research period, the center of the whiteblotched skate range showed a trend of displacement in the north direction. At the same time, the northern boundaries of its range in the Bering and Okhotsk Seas have hardly changed their position, and in the Pacific waters have noticeably shifted to the north (especially in the second decade). The center of the range in the Bering Sea had not undergone a significant shift, while in the other two areas it noticeably shifted northward. A similar pattern was observed with respect to the southern borders of the whiteblotched skate range.

***Catch dynamics*** (Figure 4 and Supplementary Figure S31). In general, a noticeable increase in catches per unit effort (more than 2 times) can be noted for the study period. The catches of whiteblotched skates in Pacific waters demonstrate a particularly sharp increase, where after a considerable decrease in their value in the third and fourth decades, there was a sharp increase. In the Bering Sea during the entire period, catches showed a progressive increase. In the Sea of Okhotsk, despite the decrease in the value of catches in recent years, the general trends showed noticeable growth.

### 3.5. Bottom Skate

**Figure 8:**
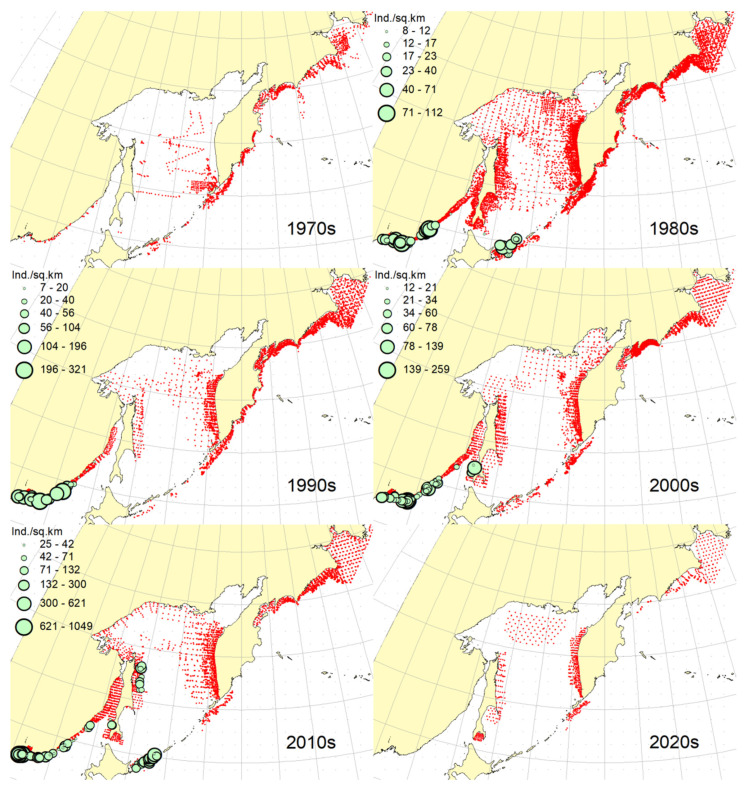
Decadal changes in the spatial distribution of the bottom skate *Bathyraja bergi* in the Northwestern Pacific. Designations as in Figure 1.

***Spatial distribution*** (Figure 8). In the 1970s and 2020s, bottom skate was not recorded in catches within the study area. In the 1980s, the main area of its occurrence and maximum catches were the northwestern Sea of Japan. The second most important area was the waters around the southern Kuril Islands. Occasional catches were recorded in the southern part of the Tatar Strait (mainland) and Sea of Okhotsk to the southeast of the Aniva Bay. In the next decade, the northwestern Sea of Japan retained its importance as the main area of occurrence and maximum catches of bottom skate. The catches in the mainland part of the Tatar Strait began to be recorded somewhat northward in comparison with the previous decade. In the 2000s, the pattern of bottom skate distribution did not fundamentally change, but catches began to occur in the eastern part of the Tatar Strait off southwestern Sakhalin. In the next decade, the range of the bottom skate significantly expanded. It reappeared in the waters of the southern Kuril Islands, and also began to occur off northeastern Sakhalin. The maximum catches during this period were recorded in the waters of the Peter the Great Bay of the Sea of Japan.

***Boundaries of the ranges*** (Figure 3, Supplementary Figures S13–S15). During decades two to five, the center of the range shifted slightly to the north on average. In the Sea of Japan, the northern border of the range has shifted noticeably to the north. The center of distribution and the southern border of the range, on the contrary, have shifted slightly southward. Data on other areas (the Sea of Okhotsk and Pacific waters) are not sufficient to judge the displacement of the boundaries of the range.

***Catch dynamics*** (Figure 4, Supplementary Figure S32). The available data allow us to correctly judge only the dynamics of bottom skate catches in the Sea of Japan. During the period under review, from the second to the fifth decade, the catches per unit effort showed an increase with some decrease in the fourth decade. In general, the catches of this species were characterized by a trend of increasing catches both in the Sea of Japan and other areas.

### 3.6. Mud Skate

**Figure 9:**
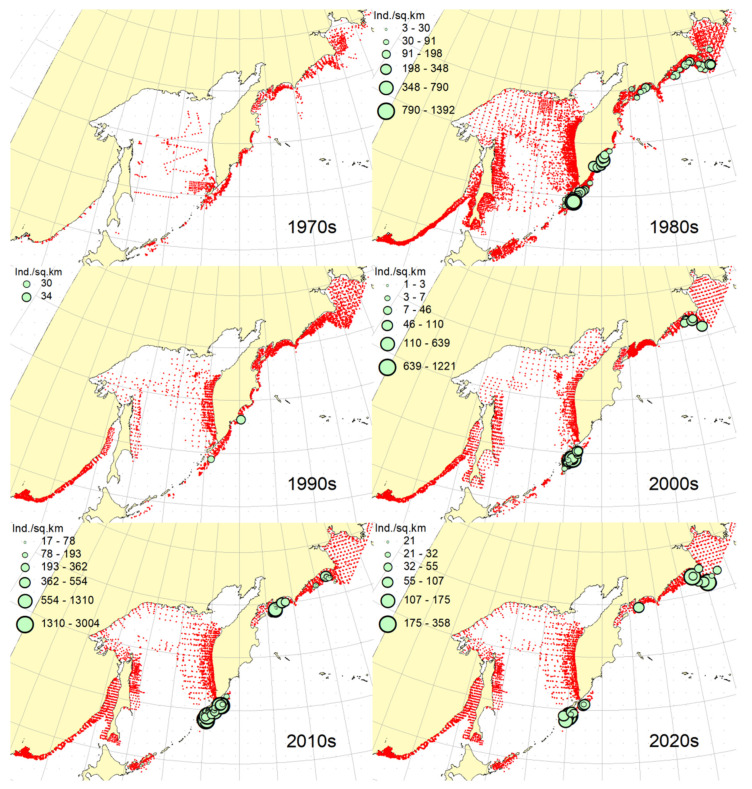
Decadal changes in the spatial distribution of the mud skate *Bathyraja taranetzi* in the Northwestern Pacific. Designations as in Figure 1.

***Spatial distribution*** (Figure 9). In the 1970s, the mud skate was not observed in the catches. During the second decade, this species was recorded in catches from the northern Kuril Islands in the south to the southern Gulf of Anadyr in the north. The maximum catches were recorded in the Pacific waters of the northern Kuril Islands and the bays of Eastern Kamchatka. In the 1990s, the mud skate was observed by occasional catches in the Pacific waters of the northern Kuril Islands and southeastern Kamchatka. In the next decade, the Pacific waters of the northern Kuril Islands again became the main area of occurrence and maximum catches. The second most important area was the western Bering Sea to the east of Navarin Cape. In the 2010s, the pattern of the distribution of catches in comparison with the previous period practically did not change. The Pacific waters of the northern Kuril Islands remained the main area of occurrence and maximum catches. However, in the western Bering Sea, large catches were recorded in the northern part of the Karagin Bay and Olyutor Bay. In recent years, the character of the spatial distribution has hardly changed, but in the western Bering Sea, the main catches have shifted to the area of Navarin Cape.

***Boundaries of the ranges*** (Figure 3, Supplementary Figures S16–S18). In general, the center of the range of the mud skate showed a southward shift from the second to the third decade, after which it shifted to the north. At the same time, the northern borders and distribution centers in the Bering Sea and Pacific waters showed trends of displacement to the south. Additionally, the trend of displacement of the southern boundary of the range in Pacific waters had a southward direction, while in the Bering Sea it was directed in the opposite direction.

***Catch dynamics*** (Figure 4, Supplementary Figure S33). Catches per unit effort of mud skate both in the Bering Sea and Pacific waters showed similar dynamics, i.e., increasing in the second, fourth and fifth decades and a fall in the third and sixth decades. Nevertheless, trends showed an increase in the value of catches per unit effort during the entire research period, both for each area and for all areas combined.

### 3.7. Whitebrow Skate

**Figure 10:**
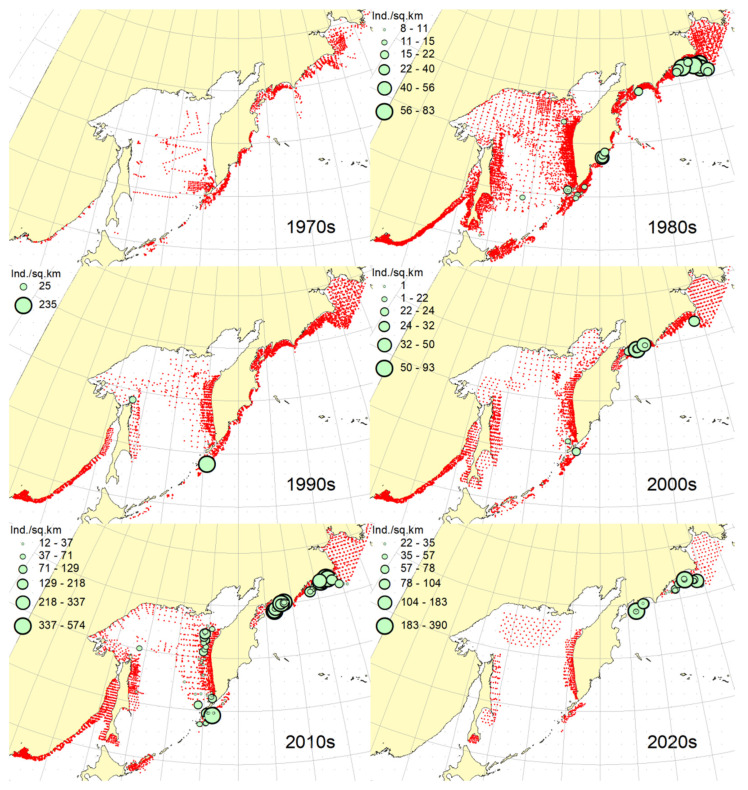
Decadal changes in the spatial distribution of the whitebrow skate *Bathyraja minispinosa* in the Northwestern Pacific. Designations as in Figure 1.

***Spatial distribution*** (Figure 10). In the first decade, catches of the whitebrow skate in the research area were not registered. In the 1980s, the area of maximum occurrence and catches was the western Bering Sea from the central part of the Koryak coast to Navarin Cape. The second most important area was the Kronotsk Bay. Single captures of this species have been recorded in the Olyutor Bay, off northwestern and southwestern Kamchatka, the central part of the Sea of Okhotsk and the Pacific waters of the northern Kuril Islands. In the 1990s, the whitebrow skate was recorded only in a single catch in the Pacific waters of the northern Kuril Islands to the east of Onekotan Island. In the 2020s, the pattern of its distribution was close to that of the 1980s, but the maximum occurrence and catch rates were characteristic of the Olyutor and Karagin Bays of the Bering Sea. In the 2010s, the range of the whitebrow skate has significantly expanded, and the number of catches has increased markedly. In addition to the western Bering Sea, which remained the main area of occurrence and maximum catches, this species began to be frequently observed along the entire Western Kamchatka and in the Pacific waters of the northern Kuril Islands. Also, its single capture was registered to the northeast of Sakhalin. In recent years, the whitebrow skate had been recorded only in the western Bering Sea from the northern part of the Karagin Bay to Navarin Cape.

***Boundaries of the ranges*** (Figure 3, Supplementary Figures S19–S21). From the second to the third decade, as a whole, a significant southward shift of the center of the range of the whitebrow skate was observed, after which it consistently and significantly shifted to the north. The northern boundaries of the distribution in all areas showed a tendency to shift in a southerly direction (well expressed in Pacific waters and less noticeable in other areas). The center of the range shifted slightly to the south in the Bering Sea and more noticeably in Pacific waters, while in the Sea of Okhotsk it showed the opposite tendency. Similar trends were noted in relation to the southern borders of the range of the whitebrow skate.

***Catch dynamics*** (Figure 4, Supplementary Figure S34). The dynamics of catches per unit effort of the whitebrow skate as a whole for all areas of its occurrence showed a series of their growth (2nd and 4th decades) and fall (3rd and 5th decades). Nevertheless, all areas combined were characterized by positive trends in the increase in catches per unit effort, i.e., the strongest in the Pacific waters and less pronounced in the Bering and Okhotsk Seas.

### 3.8. Sandpaper Skate

**Figure 11:**
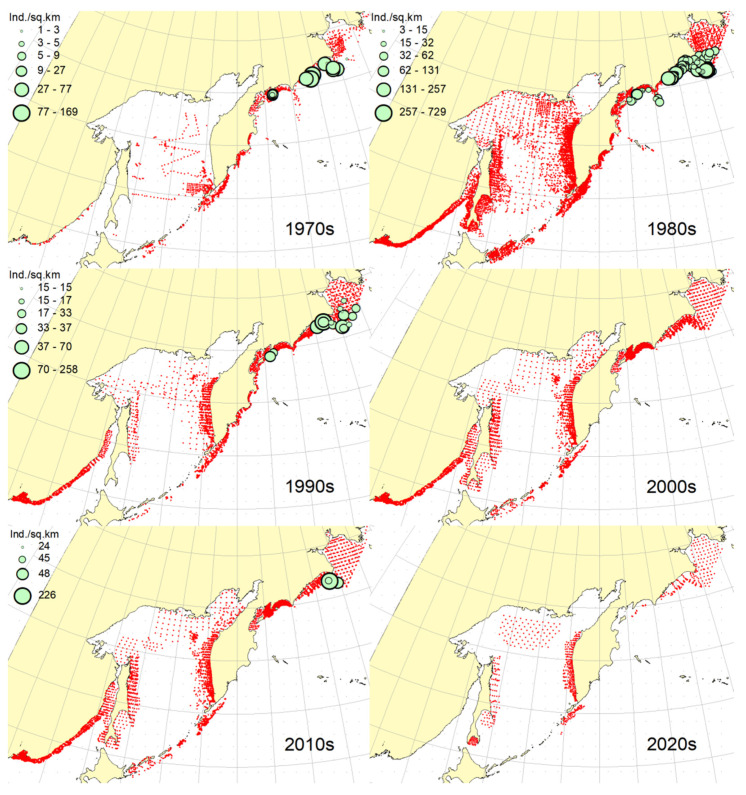
Decadal changes in the spatial distribution of the sandpaper skate *Bathyraja interrupta* in the Northwestern Pacific. Designations as in Figure 1.

***Spatial distribution*** (Figure 11). During the entire period of research, the sandpaper skate was observed in the western Bering Sea only. In the fourth and sixth decades, catches of this species in the research area were not recorded. In the 1970s, it was found in catches in the western Bering Sea from Goven Cape to Navarin Cape, and the maximum catches were recorded in the central part of the Koryak coast. In the 1980s, its number and distribution area in the western Bering Sea increased markedly. Its catches began to be observed south of Olyutor Bay in the waters of the Shirshov underwater ridge, as well as in the southern part of the Gulf of Anadyr. In the 1990s, the distribution pattern of the sandpaper skate in the western Bering Sea did not undergo significant changes, except for the extension of the range northward to the central part of the Gulf of Anadyr and absence of catches in the Olyutor Bay and waters of the western part of the Koryak coast. In the 2010s, the sandpaper skate was recorded in only a few catches off the Navarin Cape.

***Boundaries of the ranges*** (Figure 3, Supplementary Figures S22–S24). The fragmentary nature of the data and the short time series of observations do not allow us to judge with certainty about the dynamics of the position of sandpaper skate range boundaries. In general, we can only note a trend towards a southward shift of the northern border and a trend towards a northward shift of the center of the range and its southern border.

***Catch dynamics*** (Figure 4 and Supplementary Figure S35). It is difficult to judge the dynamics of sandpaper skate catches in the western Bering Sea due to the lack of a continuous series of data. Nevertheless, the trend of catch per unit effort during the study period demonstrated their sharp decline.

### 3.9. Challenger’s Skate

**Figure 12:**
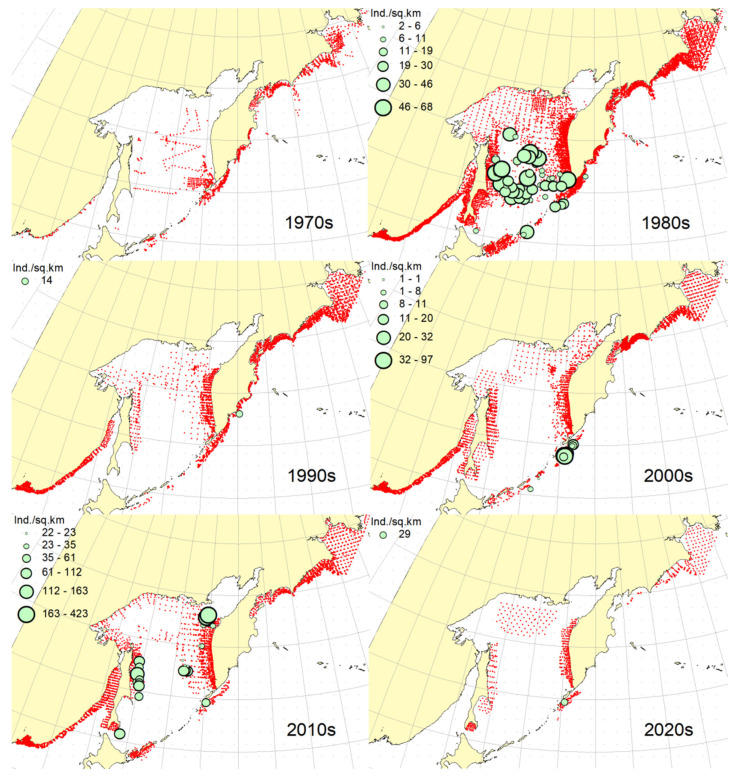
Decadal changes in the spatial distribution of the Challenger’s skate *Bathyraja isotrachys* in the Northwestern Pacific. Designations as in Figure 1.

***Spatial distribution*** (Figure 12). In the first decade, there were no captures of the Challenger’s skate within the surveyed area. Occasional catches in the waters of southeastern Kamchatka and the Pacific waters of the northern Kuril Islands were recorded, respectively, in the 1990s and the current decade. This species was most widely distributed in the study area in the 1980s. During this period, it was most numerous in the Sea of Okhotsk, occurring mainly in the central part from southeastern and northeastern Sakhalin to southwestern Kamchatka. In Pacific waters, this species has been recorded in catches from Urup Island to Avacha Bay in Kamchatka. The maximum catches were recorded in the central part of the Eastern Sakhalin, southwestern Kamchatka and in the central part of the Sea of Okhotsk. The 2000s were characterized by the capture of the Challenger’s skate exclusively in the Pacific waters of the Kuril Islands from Urup Island to Paramushir Island with maximum catches eastward to Onekotan Island. In the 2010s, catches of this species in the Pacific waters of the Kuril Islands were occasional, and its greatest occurrence was observed off Eastern Sakhalin and Western Kamchatka. The maximum catches were recorded in the northern Sea of Okhotsk off the coast of northwestern Kamchatka.

***Boundaries of the ranges*** (Figure 3, Supplementary Figures S25–S27). In general, the center of the range of the Challenger’s skate was characterized by a series of shifts to the north (2nd and 4th decades) and south (3rd and 5th decades). At the same time, the most complete data are available only for Pacific waters. The northern border of the range in this area showed a well-marked trend of southward displacement; the center of the range also shifted to the south (but less sharply), and the southern border of the range, on the contrary, shifted to the north.

***Catch dynamics*** (Figure 4, Supplementary Figure S36). Generalized data for all areas showed that Challenger’s skate catches per unit effort increased during the study period, with the exception of recent years. Nevertheless, in general, they showed a positive trend. In the Pacific waters and Sea of Okhotsk (despite the limited data in the latter area), catches showed very similar long-term dynamics in increasing catches during the entire study period.

## 4. Discussion

### 4.1. Spatial Distribution

An analysis of long-term changes in the spatial distribution of nine studied species showed that in the 1970s only three species were recorded in catches: Okhotsk, Aleutian and sandpaper skates. We see the reason for this situation as follows. Two species (whiteblotched and whitebrow) were first described only in the late 1970s [52] while bottom and mud skates were described several years later [53]. However, two species described much earlier, Challenger’s skate at the end of the 19th century [54] and dusky-purple skate in the middle of the 20th century [55], were also not recorded in catches. The reason for this, in our opinion, was the lack of field guides to skates, which were developed and published only in the early 1980s [46]. This did not allow species identification during surveys in the 1970s, and therefore all rays caught were identified only to the genus level. Additionally, data for the first decade include survey results for only one year for the Sea of Japan (1978) and three years (1977–1979) for the remaining areas. Therefore, they should hardly be considered as representative ones.

The vast majority of species differed in the widest distribution within the surveyed water area in the 1980s. The most likely reason for this is the specifics of surveys. During this period, research funding was at its maximum. The research fleet consisted of dozens of vessels, including medium- and large-tonnage ones, capable of bottom trawling to a depth of about 2 km [37,56,57]. This period was characterized by the maximum number of stations, surveyed depths and areas. All this, in our opinion, led to the most complete account of skates within the study area.

In the 1990s, the surveyed area, number of trawl stations (more than 2 times) and range of surveyed depth significantly decreased. This was due to the collapse of the former USSR and economic crisis that followed when research funding was significantly reduced. In the 2000s, with the improvement of the economic situation in Russia, the amount of funding for scientific research increased slightly, which made it possible to significantly increase the number of stations and expand surveyed areas. However, the range of surveyed depths noticeably narrowed, which affected the occurrence of skates in catches. In the 2010s, the number of hauls compared with the previous period hardly changed. However, the range of surveyed depths became even narrower, which resulted in a large degree of underestimation of skates in the study area. Additionally, it should be noted that in the fourth and fifth decades, the total surveys were not carried out in the waters of Eastern Kamchatka, or in the fifth decade in the Pacific waters of the central Kuril Islands. The data for the last (sixth) decade cannot be considered representative since they were obtained for only two years (2020–2021).

Thus, the nature of the data presented by us on the spatial distribution of nine species of deep-sea skates within the Russian EEZ in the northwestern Pacific is largely due to some artifacts. However, there are no other such detailed and long-term observations. The value of the presented data lies in the fact that, despite their scarcity, they still make it possible to present the nature of the spatial distribution and its long-term changes in nine species of skates in the study area, to determine the areas of their main concentrations based on the catches and assess the fishing potential.

Until recently, the features of the spatial distribution of the Okhotsk skate were described in general terms only for the Pacific waters of the northern Kuril Islands and southeastern Kamchatka [17,29]. The distribution of this species throughout the North Pacific, including the waters of Russia, is reviewed in a recent publication [33]. Despite the fact that the same value (ind./km^2^) was used to characterize the distribution of catches in our and the mentioned work, the data on the long-term dynamics of the distribution turned out to be difficult to compare, since different time intervals were used and the visual representation of the features of the distribution of species varies greatly.

Data on the spatial distribution of the Aleutian, whiteblotched, whitebrow, mud, dusky-purple and Challenger’s skates have so far been limited only to information on the occurrence and general features of the localization of catches and their magnitude in the Pacific waters of the northern Kuril Islands and southeastern Kamchatka, obtained in the 1990s [17,29,30,31]. Data on the distribution of the sandpaper skate in the northwestern Pacific have not yet been presented in the literature. Therefore, this article significantly expands the understanding of the occurrence, size of the catches, features of the spatial distribution and its long-term changes of the nine most common species of deep-sea skates within the Russian EEZ

### 4.2. Boundaries of the Ranges

Nowhere in any species is there a monotonous shift of the northern or southern border of the range or its center for the entire observation period in one direction—only to the south or only to the north. The shifts alternate in a different order. At the same time, positive and negative shifts in different species in different water bodies occur in different decades (Table 3). It can be said that the ranges slowly fluctuate or pulsate near some average long-term positions.

We tried to evaluate the results of the six-decade shifts of all changes using regression analysis. Despite the fact that this analysis revealed some trends in the shifts of the boundaries and centers of ranges in all nine species studied (see Supplementary Materials), statistically significant changes are observed only in a small number of cases (last column in Table 3). Thus, the northern boundary of the range of the Okhotsk skate has significantly shifted to the north in the Bering Sea and, on the contrary, to the south in Pacific waters. The center of the range of this species in the Sea of Okhotsk has shifted to the north. The distribution centers of the Aleutian and whiteblotched skates in the Sea of Okhotsk have significantly shifted to the north. The southern boundaries of the dusky-purple skate range in the Sea of Okhotsk and Pacific waters have also shifted northward. The reasons for such shifts in ranges are still unclear, although, most likely, they are due to climate change. However, finding links between changes in range and climate (oceanological conditions) requires additional research and will probably be the subject of a separate future publication. Only then will it be possible to understand whether the observed ranges’ fluctuations are random or associated with some external factors.

### 4.3. Catch Dynamics

Until recently, the catch dynamics of the Okhotsk skate were considered [17] for the Pacific waters of the northern Kuril Islands and southeastern Kamchatka, and only for a time period limited to eight years from 1993 to 2000. The dynamics of this species’ catches in various areas of the North Pacific, including the waters of Russia, are also presented in a recent publication [33]. If in our and the mentioned works the trends of catches per effort of this species in the western part of the Bering Sea coincide, then in the Sea of Okhotsk and Pacific waters they show the opposite pattern. The reason for such differences, in addition to different primary data, may be a different calculation method. Unfortunately, the above-mentioned publications do not provide a statistical analysis of the calculated trends.

Data on the dynamics of catches of the Aleutian, whiteblotched, whitebrow, mud and dusky-purple skates have so far been limited only to information on changes in the magnitude of catches in the Pacific waters of the northern Kuril Islands and southeastern Kamchatka, obtained in 1993–2000 [17,31]. Data on long-term changes in the catches of the sandpaper skate in the northwestern Pacific have not yet been presented in literature. Therefore, the data presented by us on the long-term dynamics of catches of the nine most common species of deep-sea skates within the Russian EEZ will help in the future to better understand the reasons that determine the fluctuations in the abundance of not only skates of the genus Bathyraja, but also, possibly, other deep-sea fish species, which today remain poorly understood from various points of view [58].

Despite the fact that the analysis performed made it possible to identify certain trends in the dynamics of catches in all nine species studied during the study period, statistically significant estimates were obtained only in a small number of cases (Table 4). Thus, for seven of the nine studied species, statistically significant trends in the increase in catches were revealed. For the Okhotsk and whiteblotched skates, a positive trend was noted in the Bering Sea, for the Aleutian and dusky-purple rays, in the Bering Sea and in general throughout the study area. For mud and Challenger’s skates, a similar trend was found in Pacific waters and in the study area as a whole; for whitebrow skate, only in Pacific waters. The reasons for such dynamics of catches are still unclear, but, as in the case of the displacement of the range boundaries, they are probably associated with climate change. However, establishing links between catch dynamics and climate change requires additional data and research. Perhaps this will be the subject of a separate publication in the future.

However, judging by Table 4—where pluses over minuses absolutely predominate in the third and sixth columns (30 vs. 1 and 31 vs. 0, respectively); in the fourth and seventh column, minuses predominate over pluses (22 vs. 9 and 8 vs. 23, respectively); and in the fifth, their number is the same (14)—it can be concluded that in general, from the 1970s to 1980s, the number of skates increased; from the 1980s to 1990s, it decreased; from the 1990s to 2000s, it fluctuated at the achieved level; from the 2000s to 2010s, it increased again, and; from the 2010s to 2020s, it again decreased. These trends coincide with previously identified regime shifts associated with rearrangements in the abundance of the most common species under the influence of climatic and oceanological factors [59,60,61,62,63]: the 1980s are considered the era of high bio- and fish productivity in the northwestern Pacific, 1991–1995—the lowest, the second half of the 1990s—a period of recovery, and 2000s—of high productivity again. This correlation seems plausible in connection with the absence of targeted fishing for skates in the study area noted in the Introduction, because in its absence, the abundance of populations is regulated by natural causes.

The decline in skate abundance from 2010s to 2020s may be associated both with the beginning of their target fishing and an extremely small sample over the last decade—only two years 2020 and 2021 (see Table 2). This will only become clear as more information becomes available. Theoretically, in the prevailing decade, abundance changes (the predominance of pluses or minuses) could be associated with sample sizes, since their changes (see Table 1) coincide with the prevailing abundance dynamics described in the previous paragraph (see Table 4). However, it is difficult to come up with a rational interpretation of such a relationship, since the sample size affects the accuracy of the estimate of the mean, but not its value.

It is also noteworthy that among the long-term trends in the abundance of skates for all decades, which were estimated by regression equations (Supplement), only positive ones are statistically significant. All negative trends are non-significant (see the last column in Table 4). It can be said that for six decades not a single species has been significantly affected by either fishing or climate change—not even Okhotsk skate, which has a record number of four minuses in the Sea of Okhotsk (2nd line in Table 4).

## 5. Conclusions

In this paper, for the first time, we analyzed all unique available data (from bottom trawl surveys 1977–2021) on changes in the spatial distribution and abundance of the nine most common skate species in the Russian waters of the Northwestern Pacific, where their fishery is considered promising, but still not developed. We hope that it will be useful to compare the results obtained with developments in the near future, when there is expected to be a sharp increase in fishing for these species, as well as with other areas where there is already fishing pressure on these species.

## Figures and Tables

**Table 1:** Number of records of nine skate species within the Russian EEZ, 1977–2021.

Species	Common Name	Bering Sea	Sea of Okhotsk	Sea of Japan	Pacific Ocean	All Areas Combined
*Bathyraja violacea* (Suvorov, 1935)	Okhotsk skate	578	1340	4 *	835	2757
*Bathyraja aleutica* (Gilbert, 1896)	Aleutian skate	628	260	0	265	1153
*Bathyraja matsubarai* (Ishiyama, 1952)	Dusky-purple skate	323	290	0	244	857
*Bathyraja maculata* (Ishiyama et Ishihara, 1977)	Whiteblotched skate	146	177	0	177	500
*Bathyraja bergi* (Dolganov, 1983)	Bottom skate	0	16	254	33	303
*Bathyraja taranetzi* (Dolganov, 1983)	Mud skate	125	4 *	0	168	297
*Bathyraja minispinosa* (Ishiyama et Ishihara, 1977)	Whitebrow skate	155	46	0	36	237
*Bathyraja interrupta* (Gill et Townsend, 1897)	Sandpaper skate	176	0	0	0	176
*Bathyraja isotrachys* (Günther, 1877)	Challenger’s skate	0	77	0	29	106
All species combined		2131	2210	258	1787	6386

Note: Species are sorted in descending order of total occurrence. Areas with records of particular species <15 (marked with *) excluded from the analysis.

**Table 2:** Description of the data used for the analysis of spatial distribution and relative abundance of nine skate species in the Russian waters of the northwestern Pacific, 1977–2021.

Parameter	Area	Decade						Total
		1	2	3	4	5	6	
Survey years	Bering Sea	1977–1979	1980–1989	1990–1999	2000–2008	2010–2019	2020–2021	1977–2021
	Sea of Okhotsk	1977–1979	1980–1989	1990–1999	2000–2009	2010–2019	2020–2021	1977–2021
	Sea of Japan	1978	1981–1989	1990–1999	2000–2009	2010–2019	-	1978–2019
	Pacific Ocean	1977–1979	1980–1989	1990–1998	2000–2008	2012–2019	2021	1977–2021
	Total	1977–1979	1980–1989	1990–1999	2000–2009	2010–2019	2020–2021	1977–2021
Surveyed depths, m	Bering Sea	10–1150	14–1400	20–1000	12–773	10–968	18–957	10–1400
	Sea of Okhotsk	30–1350	10–2000	10–1100	5–1100	9–981	11–459	5–2000
	Sea of Japan	23–255	14–740	5–700	5–940	5–926	-	5–940
	Pacific Ocean	20–1500	10–1860	30–1530	23–1260	20–1054	70–480	10–1860
	Total	10–1500	10–2000	5–1530	5–1260	5–1054	11–957	5–2000
Stations at depths ≥400 m, %	Bering Sea	11.4	15.4	7.0	6.5	11.8	24.3	11.5
	Sea of Okhotsk	66.5	21.4	13.2	10.8	14.6	1.7	17.4
	Sea of Japan	0.0	13.3	7.6	12.1	14.7	0.0	12.4
	Pacific Ocean	11.8	27.5	17.2	17.0	26.8	5.9	20.8
	Total	21.4	19.2	10.2	11.8	14.5	8.0	15.1
Number of stations	Bering Sea	852	2942	1760	1343	1617	235	8749
	Sea of Okhotsk	358	5007	1321	2267	2931	583	12,467
	Sea of Japan	56	3212	1986	3011	3977	0	12,242
	Pacific Ocean	677	2329	982	1656	250	68	5962
	Total	1943	13,490	6049	8277	8775	886	39,420
Surveyed area, km^2^	Bering Sea	70.7	297.3	282.0	82.7	103.5	9.0	845.2
	Sea of Okhotsk	35.3	429.9	119.7	191.1	107.2	17.5	900.7
	Sea of Japan	2.9	274.8	100.0	160.0	84.1	0.0	621.8
	Pacific Ocean	71.2	295.6	236.8	753.6	9.5	2.4	1369.1
	Total	180.1	1297.5	738.5	1187.5	304.3	28.9	3736.8

Note: Surveyed area was calculated as the sum of areas covered by trawl hauls. Area of a trawl haul was calculated by multiplying trawl horizontal opening by hauling distance.

**Table 3:** Trends in shifts of the northern (max) and southern (min) boundaries of a range and its center (avg) of nine skate species in the western Bering Sea (B), Sea of Okhotsk (O), northwestern Sea of Japan (J), and Pacific Ocean (P): “+”—northward shift, positive correlation (green); “–”— southward shift, negative correlation (brown); “0”— no shift (yellow); “?”— unknown, no records of species; “ns”— non-significant correlation (yellow).

Species	Latitudinal Boundary	Area	Shift from One Decade to Another					Correlation
			1–2	2–3	3–4	4–5	5–6	
*Bathyraja violacea*	max	B	+	–	+	+	–	+
		O	+	–	+	+	–	ns
		P	+	–	+	–	–	–
	avg	B	+	–	+	+	+	ns
		O	+	+	+	–	+	+
		P	–	–	–	+	+	ns
	min	B	+	–	–	+	+	ns
		O	–	+	–	+	–	ns
		P	–	+	–	–	+	ns
*Bathyraja aleutica*	max	B	+	–	–	+	–	ns
		O	+	–	–	+	?	ns
		P	+	+	–	+	–	ns
	avg	B	–	–	–	+	–	ns
		O	+	+	–	+	?	+
		P	+	–	–	+	–	ns
	min	B	–	+	+	–	0	ns
		O	–	+	+	+	?	ns
		P	–	+	–	–	+	ns
*Bathyraja matsubarai*	max	B	?	–	+	+	–	ns
		O	?	–	–	+	?	ns
		P	?	–	+	+	–	ns
	avg	B	?	–	+	–	–	ns
		O	?	+	–	+	?	ns
		P	?	–	–	+	+	ns
	min	B	?	+	+	–	+	ns
		O	?	+	+	–	?	+
		P	?	+	–	+	+	+
*Bathyraja maculata*	max	B	?	–	+	–	–	ns
		O	?	–	+	+	–	ns
		P	+	–	–	+	–	ns
	avg	B	?	–	+	+	–	ns
		O	?	+	–	+	+	+
		P	+	–	–	+	–	ns
	min	B	?	+	+	–	+	ns
		O	?	+	–	–	+	ns
		P	–	+	–	+	+	ns
*Bathyraja bergi*	max	O	?	?	?	?	?	ns
		P	?	?	?	?	?	ns
		J	?	+	+	+	?	ns
	avg	O	?	?	?	?	?	ns
		P	?	?	?	?	?	ns
		J	?	–	+	–	?	ns
	min	O	?	?	?	?	?	ns
		P	?	?	?	?	?	ns
		J	?	–	–	–	?	ns
		P	?	–	–	+	–	ns
	avg	B	?	?	?	–	+	ns
		P	?	–	–	+	+	ns
	min	B	?	?	?	–	+	ns
		P	?	+	–	–	+	ns
*Bathyraja minispinosa*	max	B	?	?	?	+	–	ns
		O	?	–	–	+	?	ns
		P	?	–	+	+	–	ns
	avg	B	?	?	?	+	–	ns
		O	?	+	–	+	?	ns
		P	?	–	–	+	+	ns
	min	B	?	?	?	–	0	ns
		O	?	–	–	–	?	ns
		P	?	–	–	+	+	ns
*Bathyraja interrupta*	max	B	+	+	?	?	?	ns
	avg	B	+	+	?	?	?	ns
	min	B	–	+	?	?	?	ns
*Bathyraja isotrachys*	max	O	?	?	?	?	?	ns
		P	?	+	–	–	+	ns
	avg	O	?	?	?	?	?	ns
		P	?	+	–	+	+	ns
	min	O	?	?	?	?	?	ns
		P	?	+	–	+	+	ns

Note: Correlation coefficients (*r*), *p*-values, regression equations and plots are presented in the Supplementary Figures S1–S27.

**Table 4:** Trends in CPUE changes of nine skate species in the western Bering Sea (B), Sea of Okhotsk (O), northwestern Sea of Japan (J), Pacific Ocean (P), and all areas combined (T): “+”—increasing, positive correlation (green); “–”—decreasing, negative correlation (brown); “0”—no changes (yellow); “ns”—non-significant correlation (yellow).

Species	Area	From One Decade to Another					Correlation
		1–2	2–3	3–4	4–5	5–6	
*Bathyraja violacea*	B	+	+	+	+	–	+
	O	–	–	–	+	–	ns
	P	+	–	–	+	–	ns
	T	+	–	–	+	+	ns
*Bathyraja aleutica*	B	+	–	+	+	+	+
	O	+	+	+	+	–	ns
	P	+	–	–	+	+	ns
	T	+	–	+	+	+	+
*Bathyraja matsubarai*	B	+	–	+	+	+	+
	O	+	+	–	+	–	ns
	P	+	–	–	+	–	ns
	T	+	–	+	+	+	+
*Bathyraja maculata*	B	+	+	+	+	–	+
	O	+	–	+	+	–	ns
	P	+	–	–	+	+	ns
	T	+	–	–	+	+	ns
*Bathyraja bergi*	O	+	–	0	+	–	ns
	P	+	–	0	+	–	ns
	J	+	+	–	+	–	ns
	T	+	+	–	+	–	ns
*Rhinoraja taranetzi*	B	+	–	+	+	–	ns
	P	+	–	+	+	–	+
	T	+	–	+	+	–	+
*Bathyraja minispinosa*	B	+	–	+	+	–	ns
	O	+	+	–	+	–	ns
	P	+	+	–	+	–	ns
	T	+	+	–	+	–	+
*Bathyraja interrupta*	B	+	–	–	+	–	ns
*Bathyraja isotrachys*	O	+	–	0	+	–	ns
	P	+	–	+	+	–	+
	T	+	–	+	+	–	+

Note: Correlation coefficients (*r*), *p*-values, regression equations and plots are presented in the Supplementary Figures S28–S36.

## Data Availability

Not applicable.

## Supplementary Materials

The following supporting information can be downloaded at: https://www.mdpi.com/article/10.3390/ani12243485/s1, Figures S1–S27: Trends of shifts of the northern and southern boundaries of range and its center of nine skate species in the Northwestern Pacific; Figures S28–S36: Trends of CPUE changes of nine skate species in the Northwestern Pacific.
