# Supplementary material for: Long-Term Changes in the Distribution and Abundance of Nine Deep-Water Skates (Arhynchobatidae: Rajiformes: Chondrichthyes) in the Northwestern Pacific
Source: Animals (Basel). 2022 Dec 9;12(24):3485. doi: 10.3390/ani12243485 (PMC9774831; doi:10.3390/ani12243485)
Supplement: Supplementary file 1 [file animals-12-03485-s001.zip › animals-2017401-supplementary.pdf]

Long-term Changes of Distribution and Abundance of Nine Deep-water Skates (Arhynchobatidae: Rajiformes: Chondrichthyes) in the Northwestern Pacific

Alexei M. Orlov[1,2,3,4,5*], Igor V. Volvenko[6]

1 Laboratory of Oceanic Ichthyofauna, Shirshov Institute of Oceanology of the Russian Academy of Sciences, 117218 Moscow, Russia
2 Laboratory of Behavior of Lower Vertebrates, A.N. Severtsov Institute of Ecology and Evolution of the Russian Academy of Sciences, 119071 Moscow, Russia
3 Department of Ichthyology and Hydrobiology, Tomsk State University, 634050 Tomsk, Russia
4 Department of Ichthyology, Dagestan State University, 367000 Makhachkala, Russia
5 Laboratory of Marine Biology, Caspain Institute of Biological Resources of Dagestan Federal Research Center of the Russian Academy of Sciences, 367000 Makhachkala, Russia
6 Pacific Branch of the Russian Federal Research Institute of Fisheries and Oceanography, 690091 Vladivostok, Russia

* Correspondence: orlov@vniro.ru

Supplement

Figures S1-S27 – Trends of shifts of the northern and southern boundaries of range and its center of nine skate species in the Northwestern Pacific. **Figures S28-S36** – Trends of CPUE changes of nine skate species in the Northwestern Pacific.

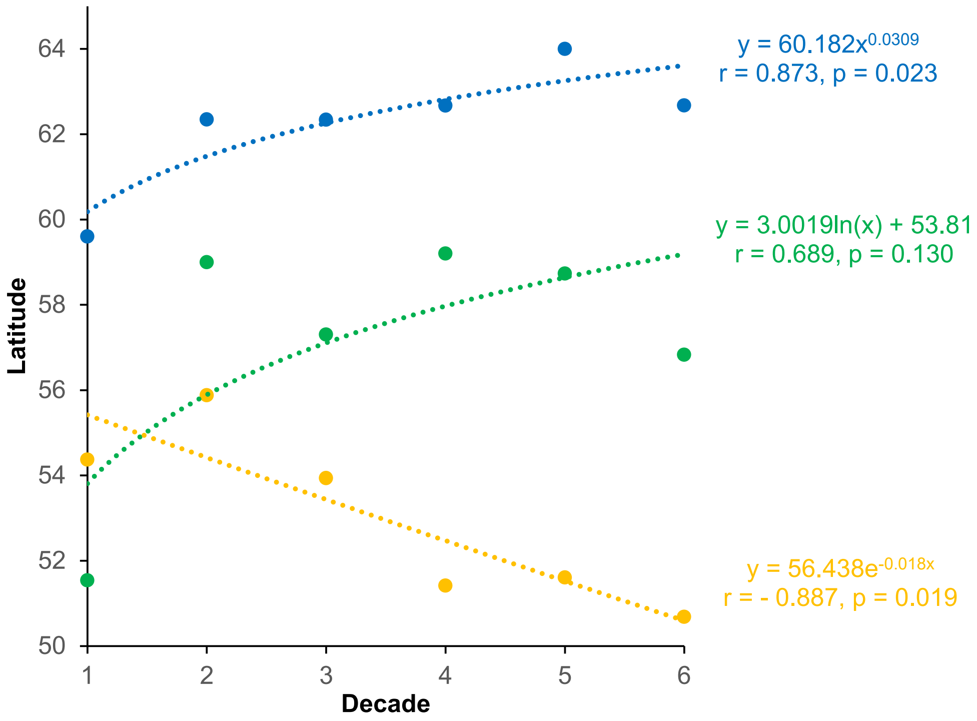

Figure S1. Trends of shifts of the northern boundaries of the Okhotsk skate *Bathyraja violacea* range in the Northwestern Pacific. Here and on the Figs. S2-S27 the Bering Sea is in blue, the Sea of Okhotsk is in green, the Sea of Japan is in red, and the Pacific Ocean is in yellow.

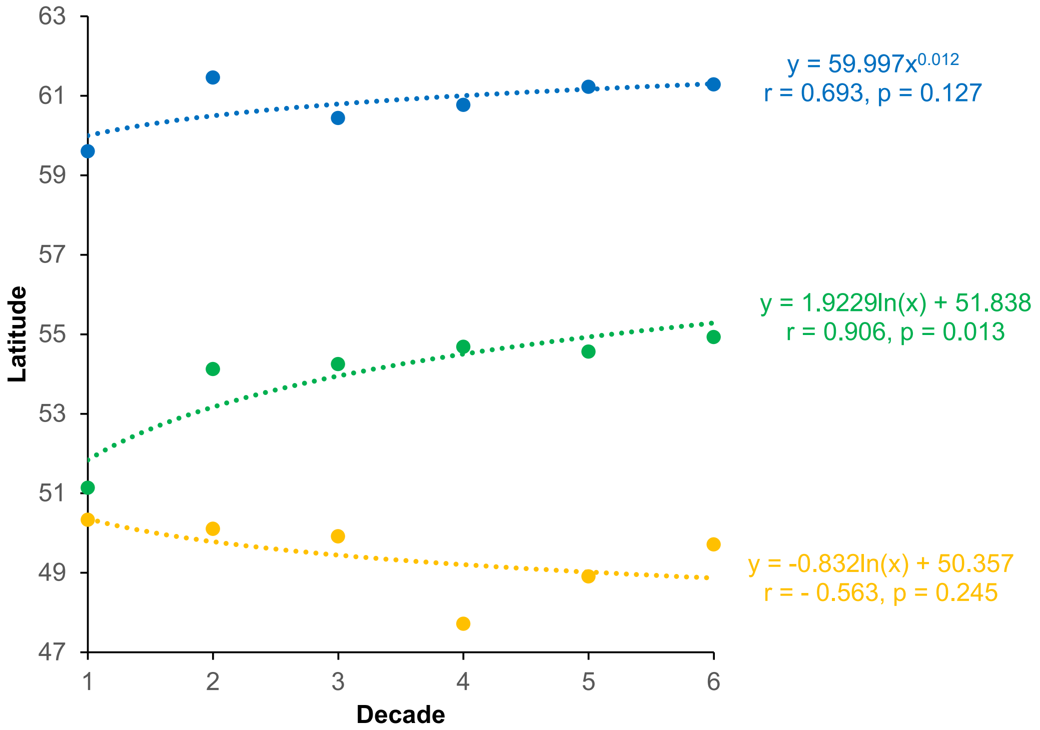

Figure S2. Trends of shifts of the center of the Okhotsk skate *Bathyraja violacea* range in the Northwestern Pacific. Designations as in Figure S1.

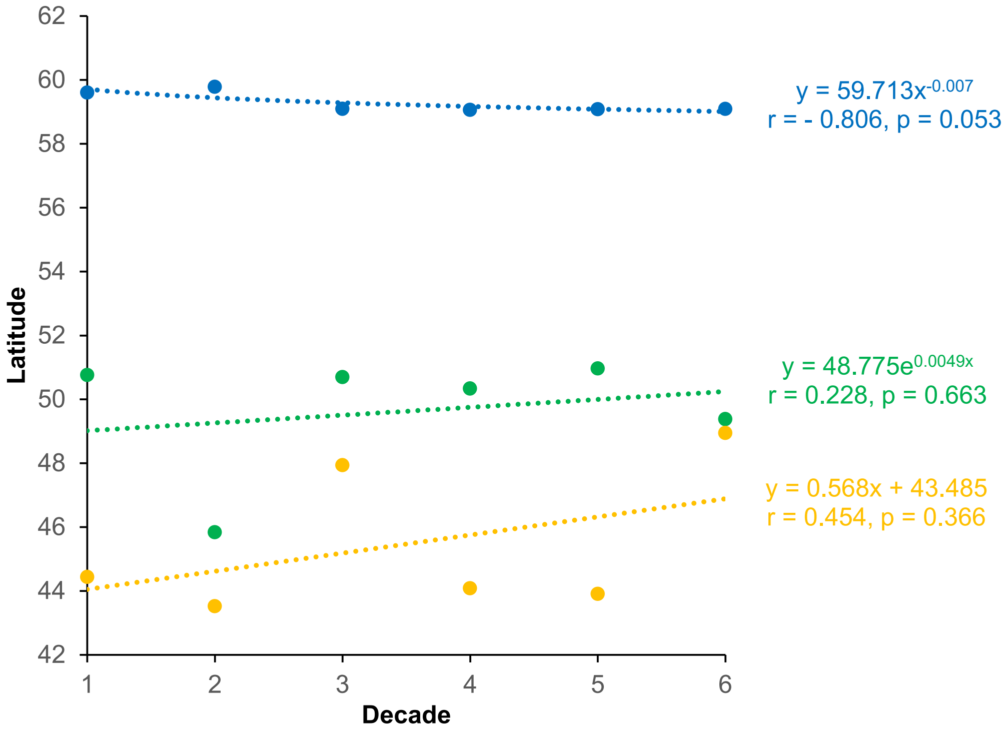

Figure S3. Trends of shifts of the southern boundaries of the Okhotsk skate *Bathyraja violacea* range in the Northwestern Pacific. Designations as in Figure S1.

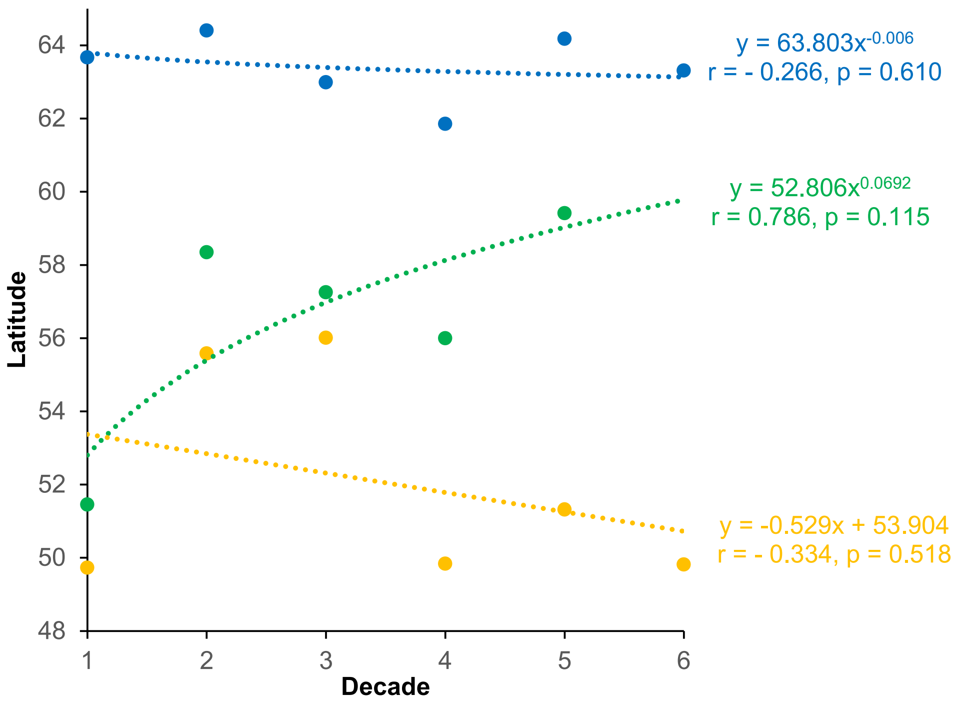

Figure S4. Trends of shifts of the northern boundaries of the Aleutian skate *Bathyraja aleutica* range in the Northwestern Pacific. Designations as in Figure S1.

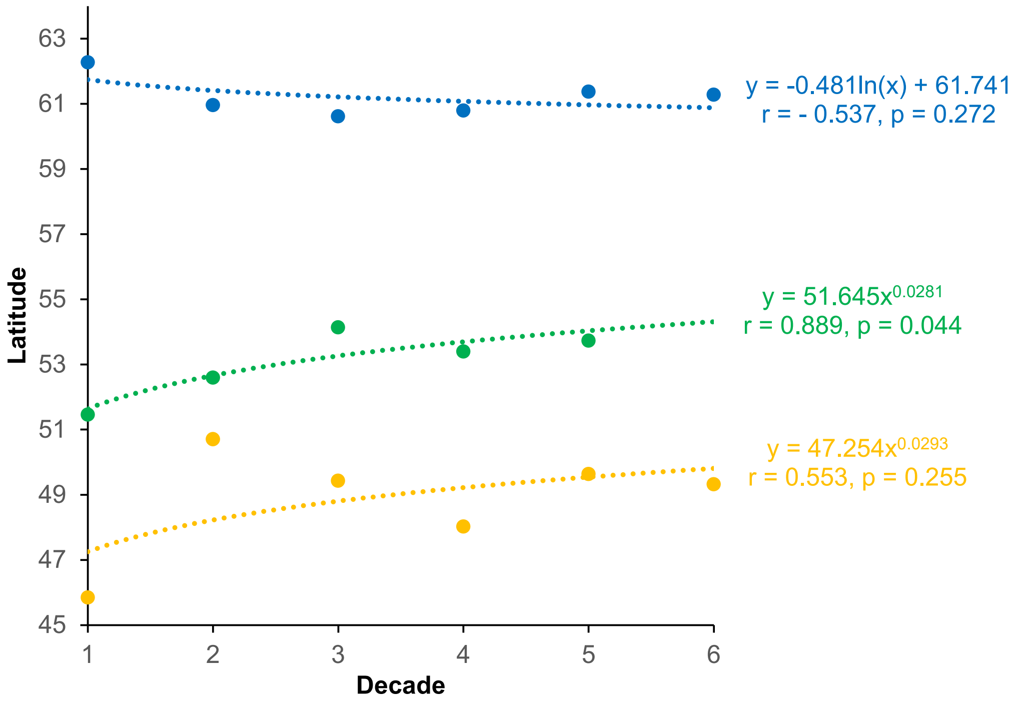

Figure S5. Trends of shifts of the center of the Aleutian skate *Bathyraja aleutica* range in the Northwestern Pacific. Designations as in Figure S1.

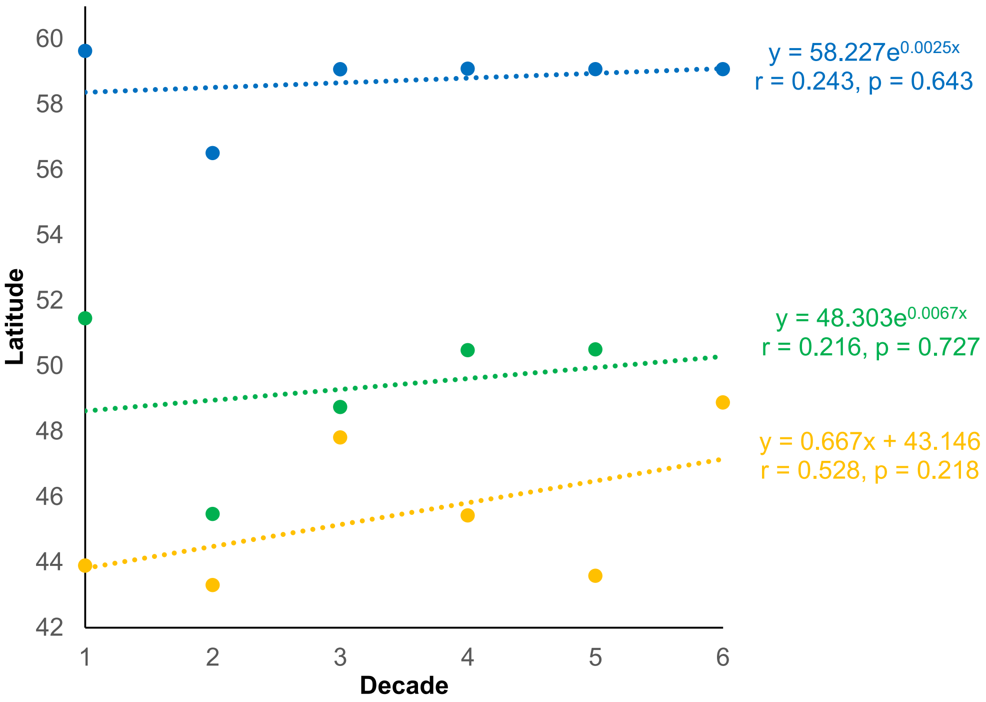

Figure S6. Trends of shifts of the southern boundaries of the Aleutian skate *Bathyraja aleutica* range in the Northwestern Pacific. Designations as in Figure S1.

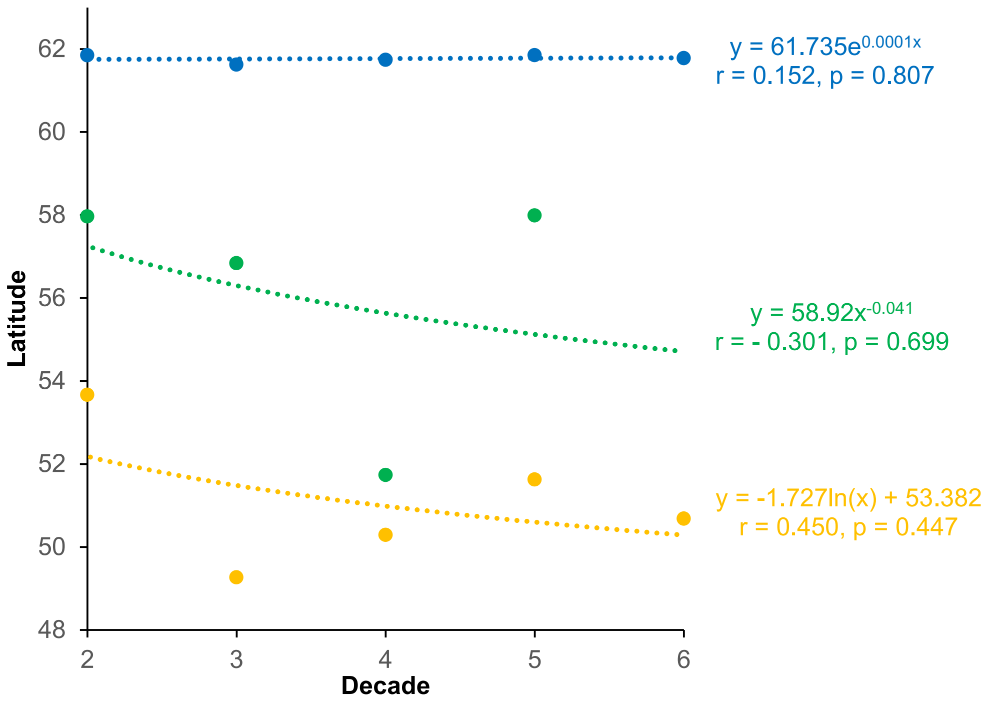

Figure S7. Trends of shifts of the northern boundaries of the dusky-purple skate *Bathyraja matsubarai* range in the Northwestern Pacific. Designations as in Figure S1.

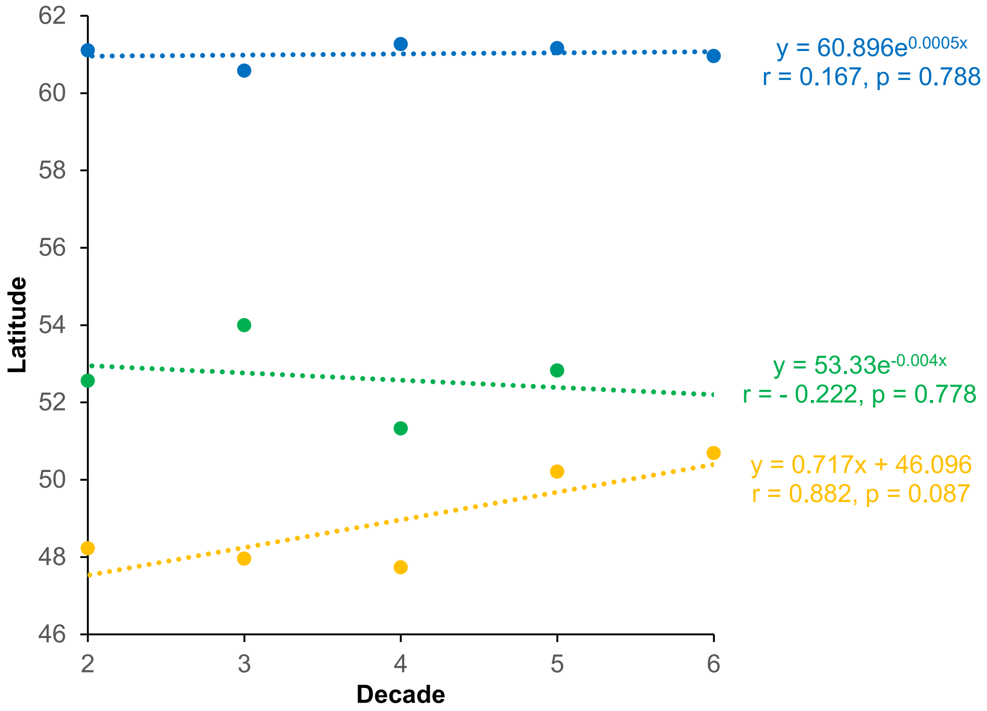

Figure S8. Trends of shifts of the center of the dusky-purple skate *Bathyraja matsubarai* range in the Northwestern Pacific. Designations as in Figure S1.

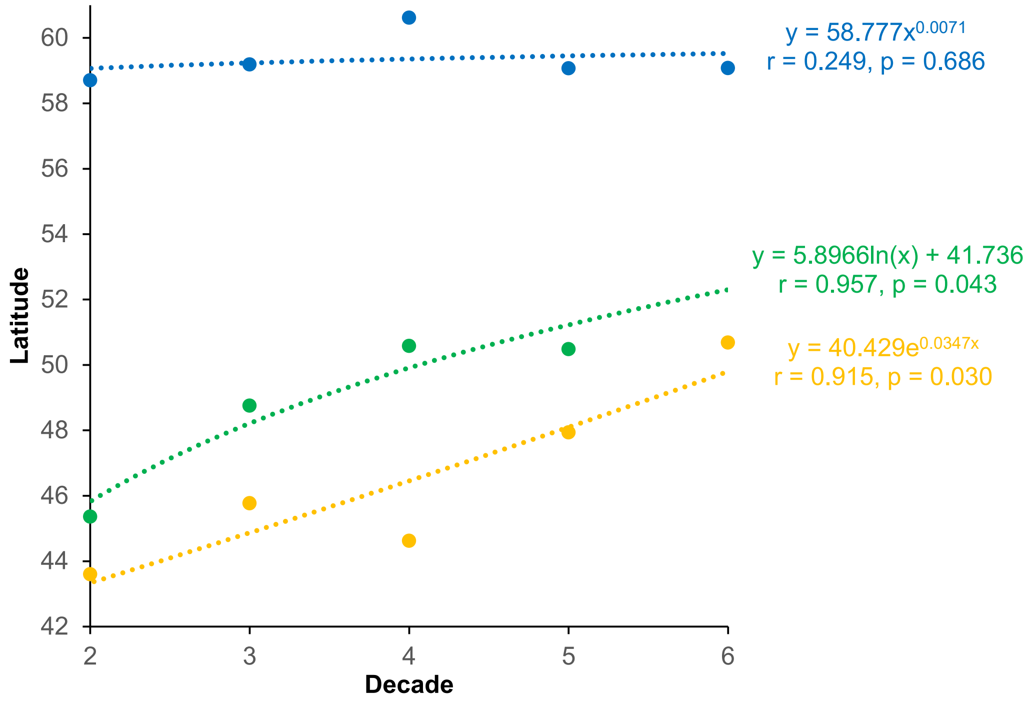

Figure S9. Trends of shifts of the southern boundaries of the dusky-purple skate *Bathyraja matsubarai* range in the Northwestern Pacific. Designations as in Figure S1.

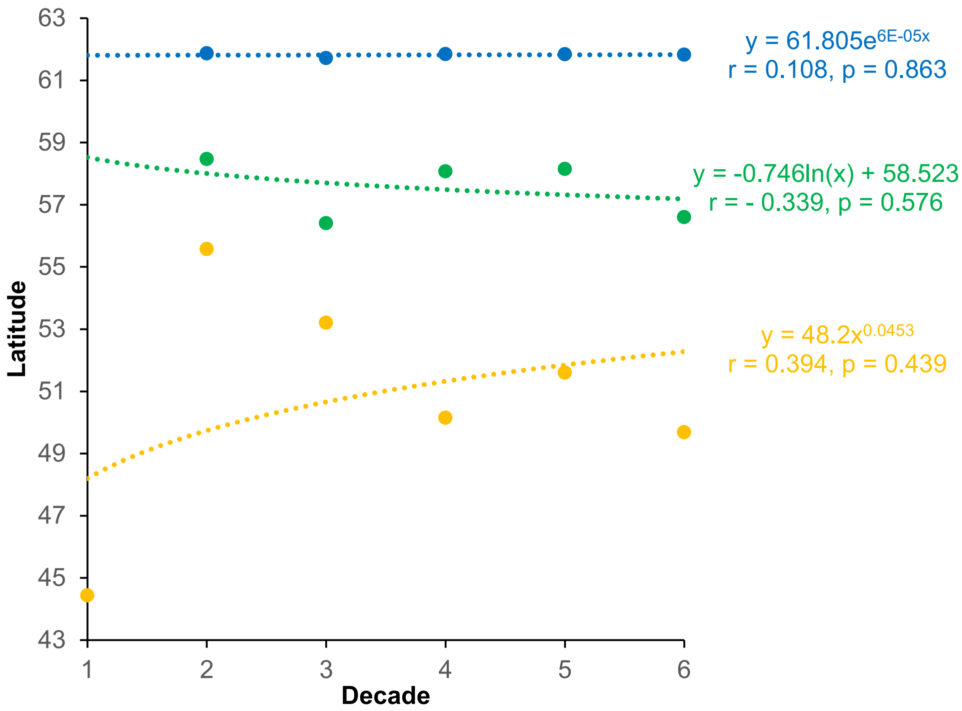

Figure S10. Trends of shifts of the northern boundaries of the whiteblotched skate *Bathyraja maculata* range in the Northwestern Pacific. Designations as in Figure S1.

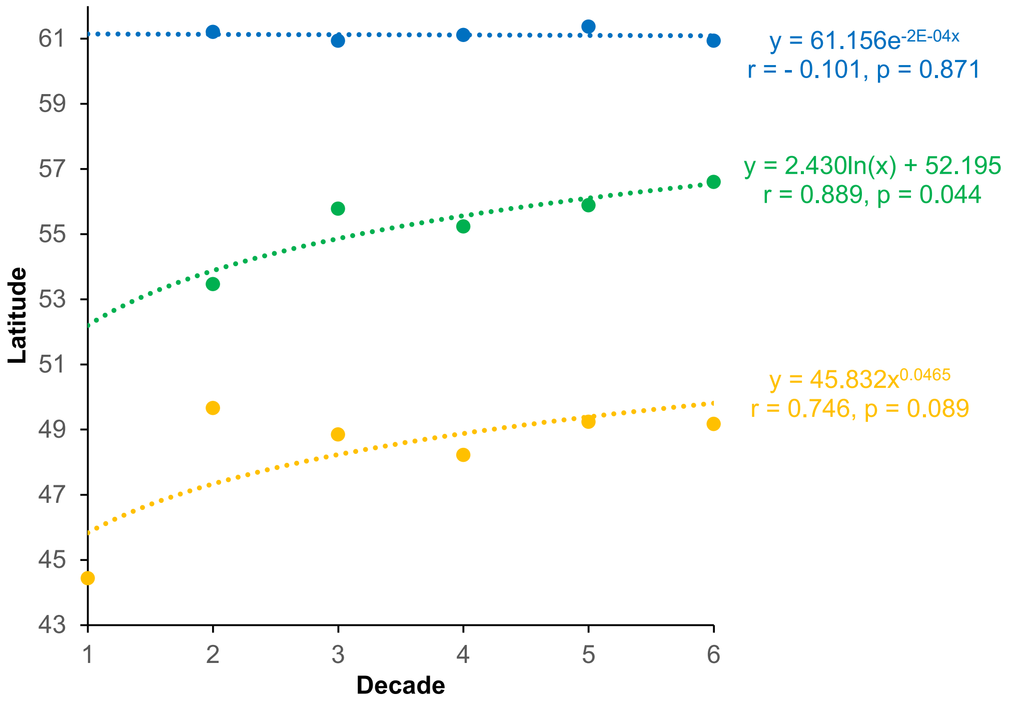

Figure S11. Trends of shifts of the center of the whiteblotched skate *Bathyraja maculata* range in the Northwestern Pacific. Designations as in Figure S1.

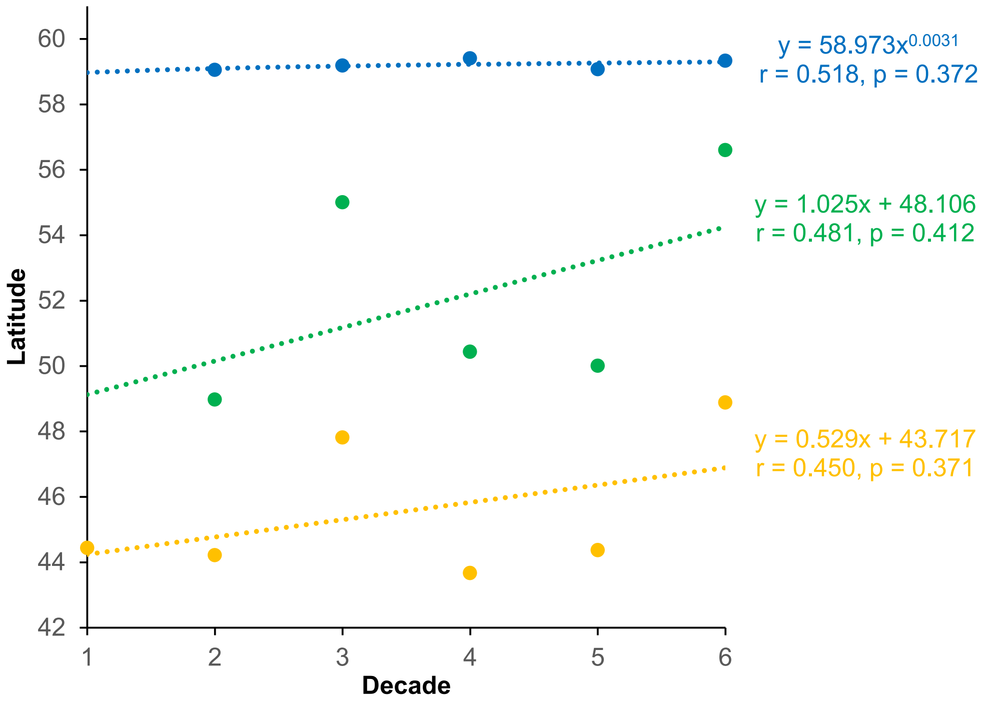

Figure S12. Trends of shifts of the southern boundaries of the whiteblotched skate *Bathyraja maculata* range in the Northwestern Pacific. Designations as in Figure S1.

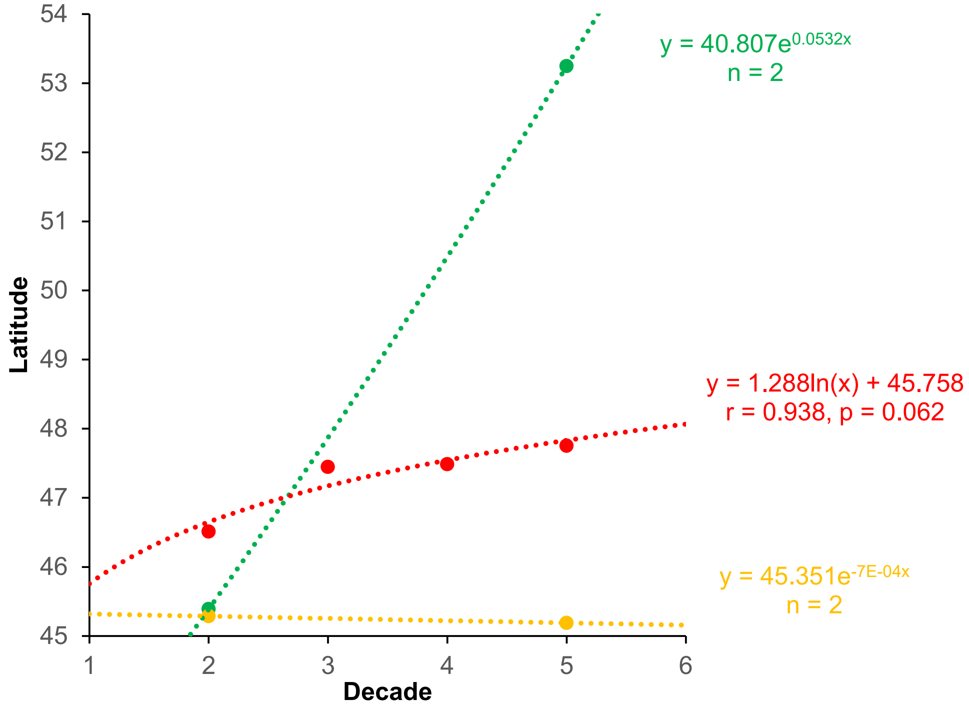

Figure S13. Trends of shifts of the northern boundaries of the bottom skate *Bathyraja bergi* range in the Northwestern Pacific. Designations as in Figure S1.

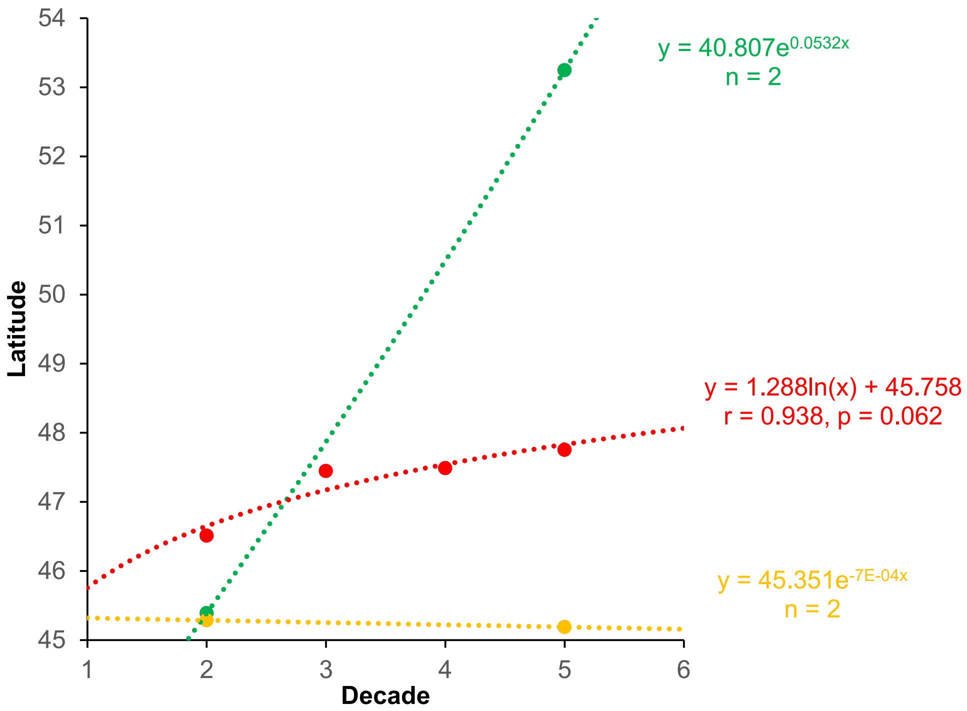

Figure S14. Trends of shifts of the center of the bottom skate *Bathyraja bergi* range in the Northwestern Pacific. Designations as in Figure S1.

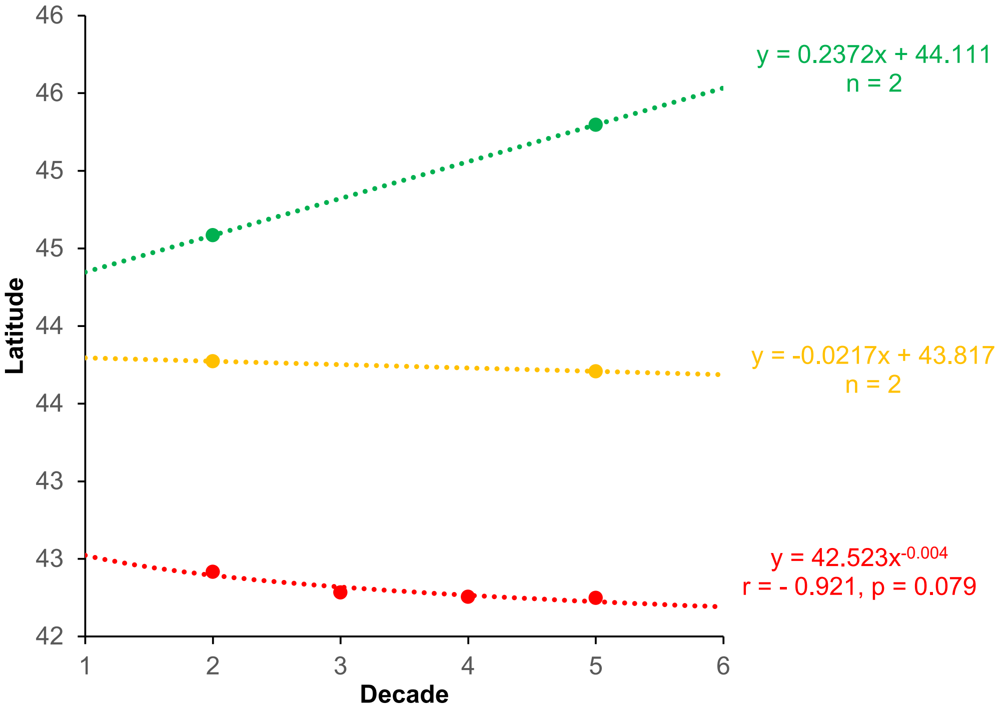

Figure S15. Trends of shifts of the southern boundaries of the bottom skate *Bathyraja bergi* range in the Northwestern Pacific. Designations as in Figure S1.

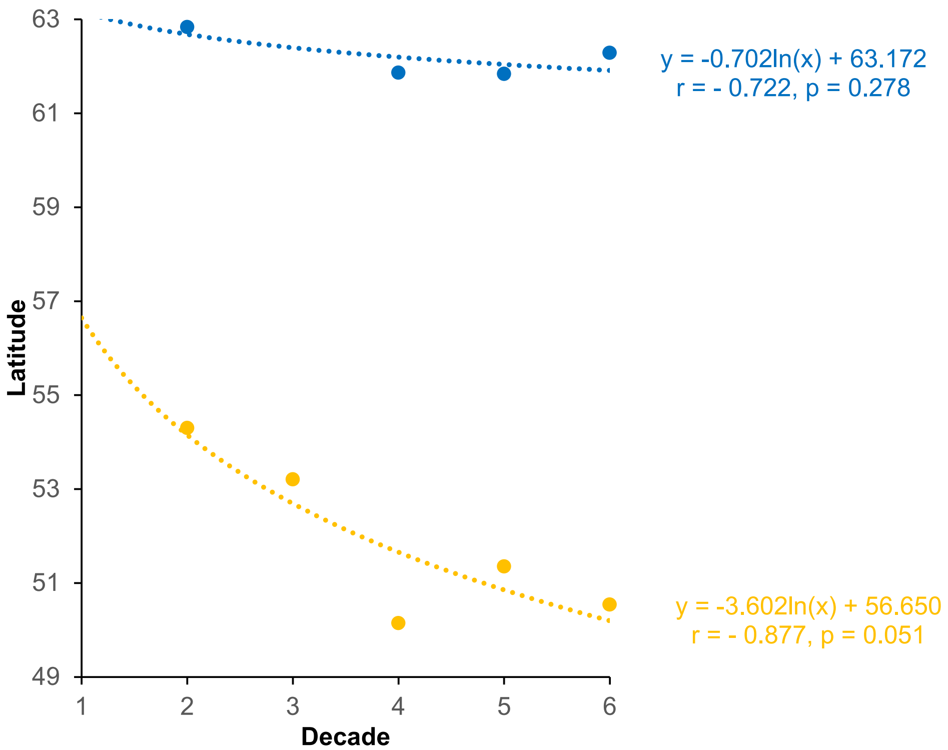

Figure S16. Trends of shifts of the northern boundaries of the mud skate *Bathyraja taranetzi* range in the Northwestern Pacific. Designations as in Figure S1.

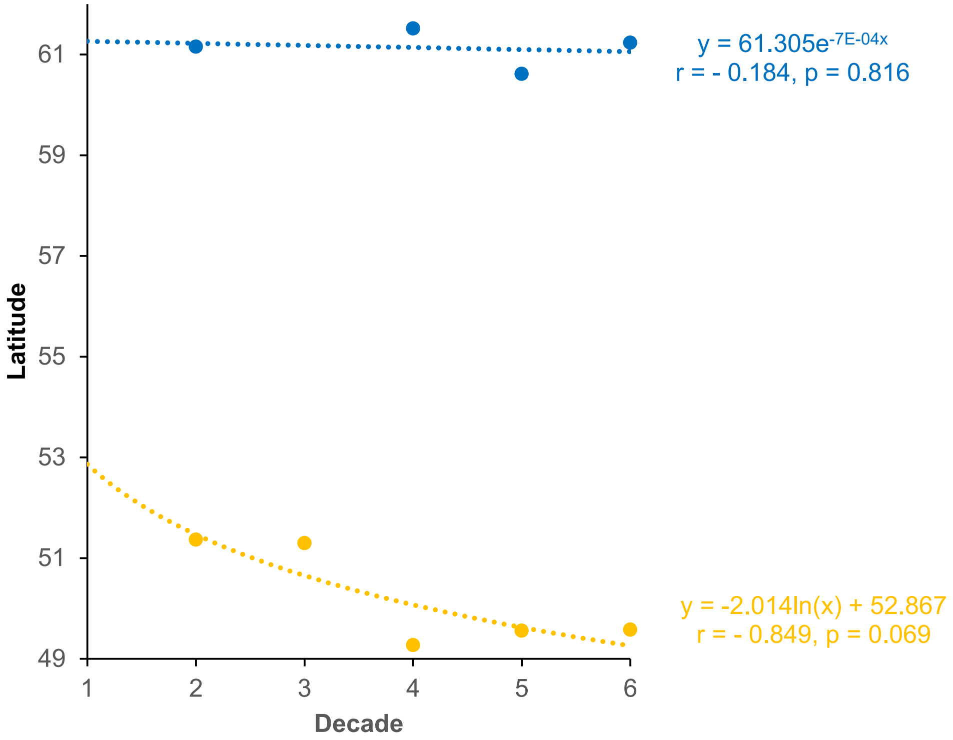

Figure S17. Trends of shifts of the center of the mud skate *Bathyraja taranetzi* range in the Northwestern Pacific. Designations as in Figure S1.

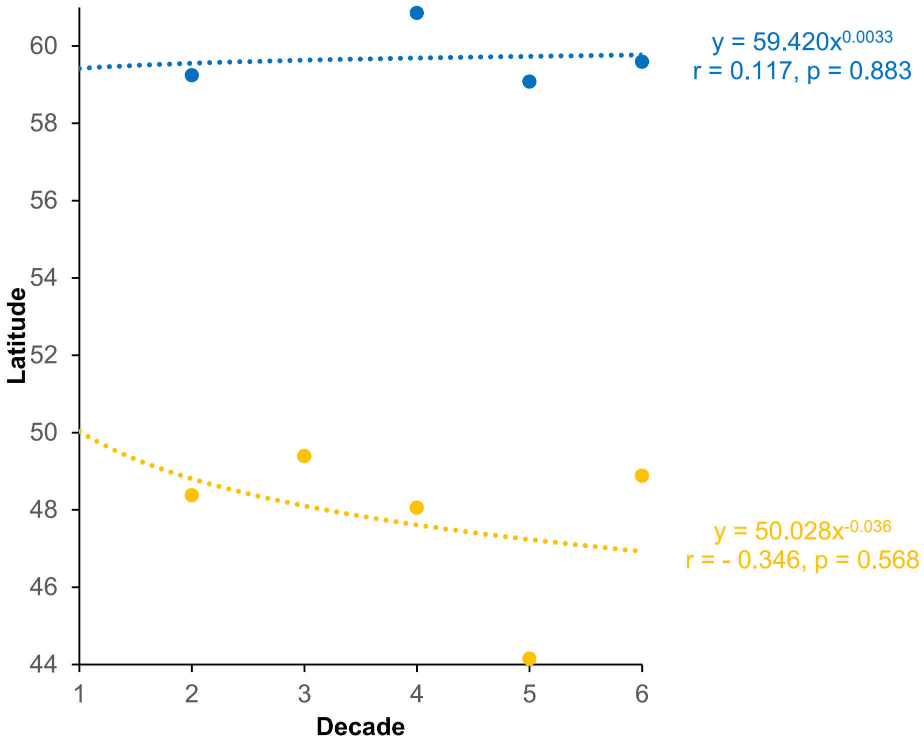

Figure S18. Trends of shifts of the southern boundaries of the mud skate *Bathyraja taranetzi* range in the Northwestern Pacific. Designations as in Figure S1.

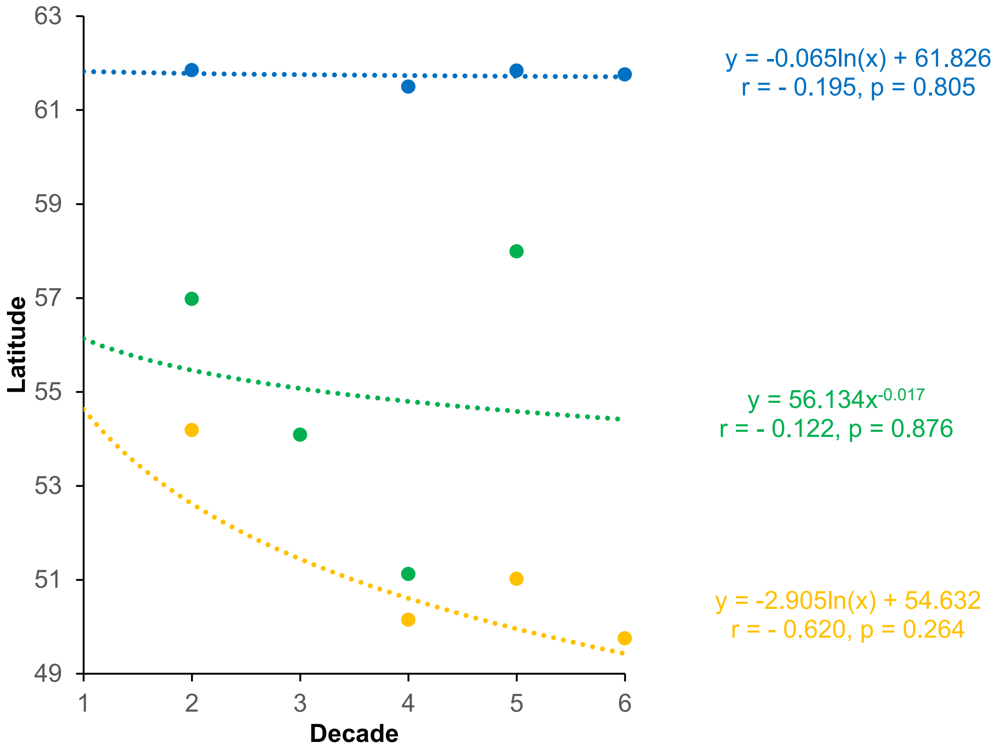

Figure S19. Trends of shifts of the northern boundaries of the whitebrow skate *Bathyraja minispinosa* range in the Northwestern Pacific. Designations as in Figure S1.

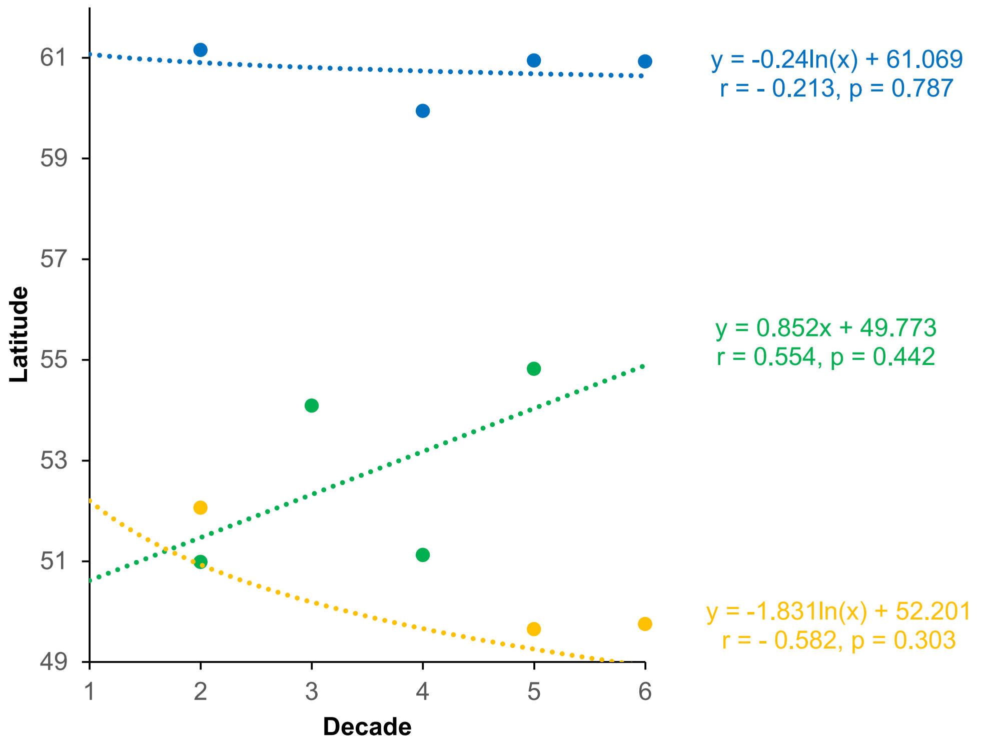

Figure S20. Trends of shifts of the center of the whitebrow skate *Bathyraja minispinosa* range in the Northwestern Pacific. Designations as in Figure S1.

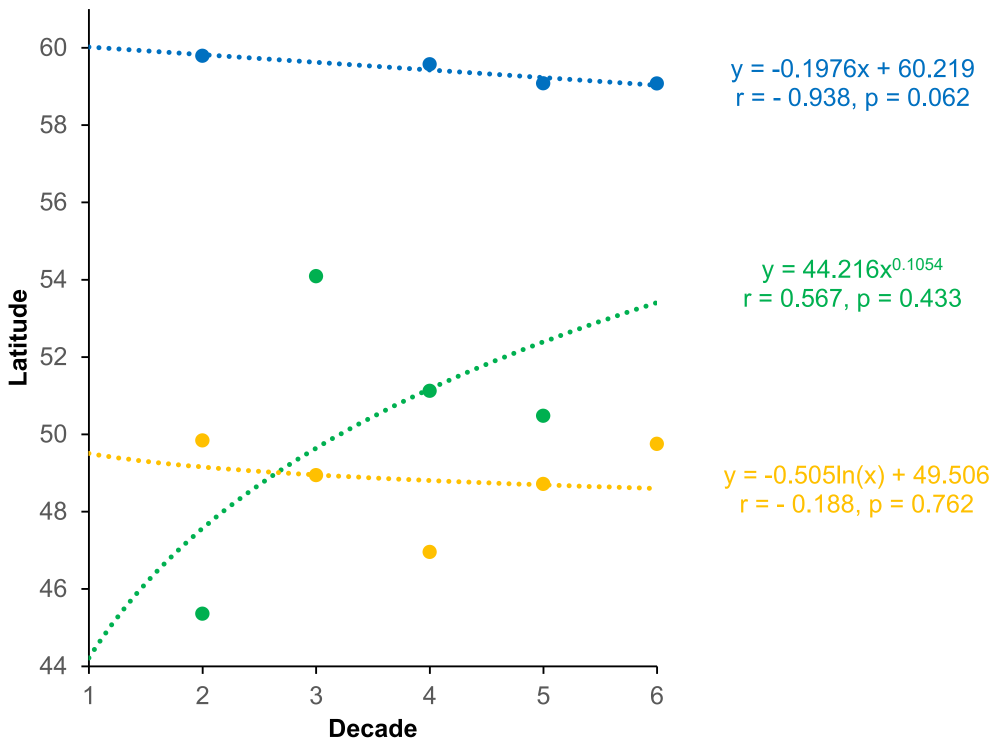

Figure S21. Trends of shifts of the southern boundaries of the whitebrow skate *Bathyraja minispinosa* range in the Northwestern Pacific. Designations as in Figure S1.

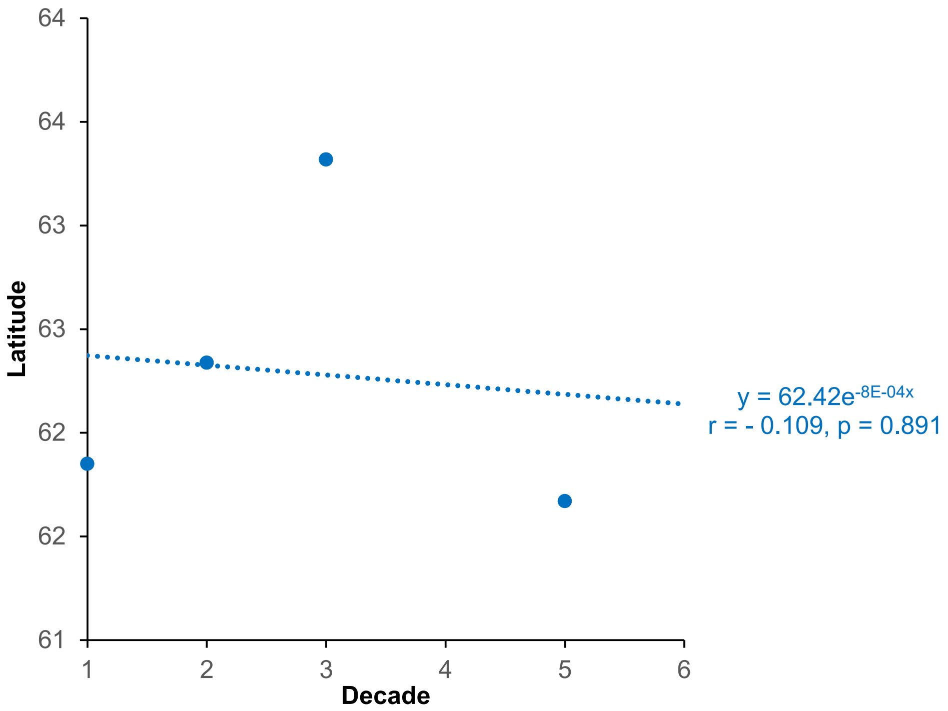

Figure S19. Trends of shifts of the northern boundaries of the sandpaper skate *Bathyraja interrupta* range in the Northwestern Pacific. Designations as in Figure S1.

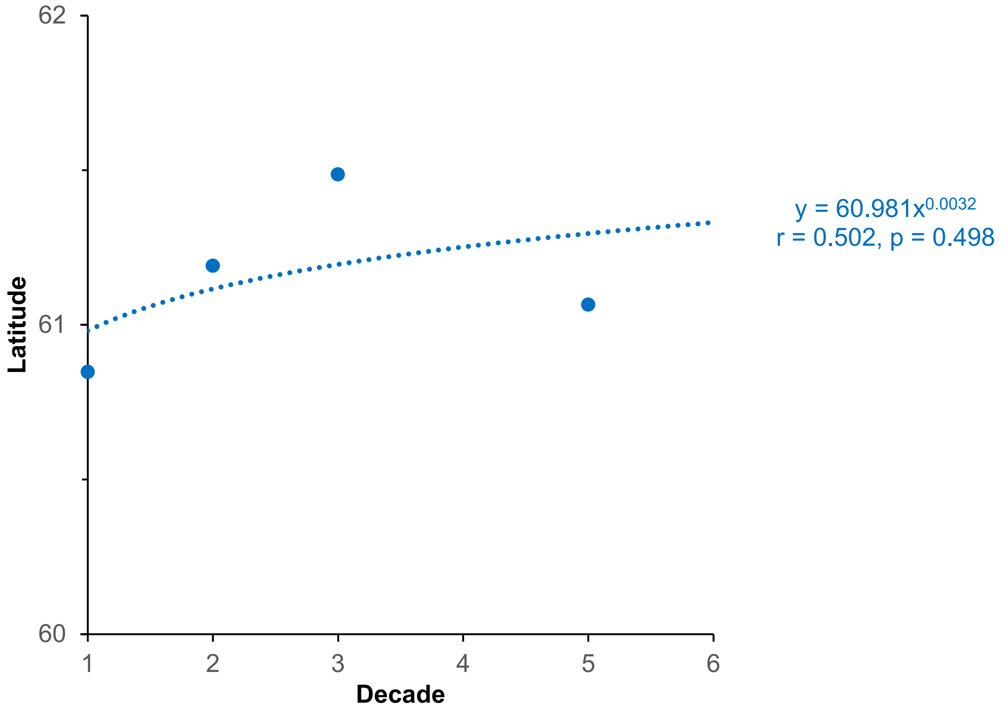

Figure S23. Trends of shifts of the center of the sandpaper skate *Bathyraja interrupta* range in the Northwestern Pacific. Designations as in Figure S1.

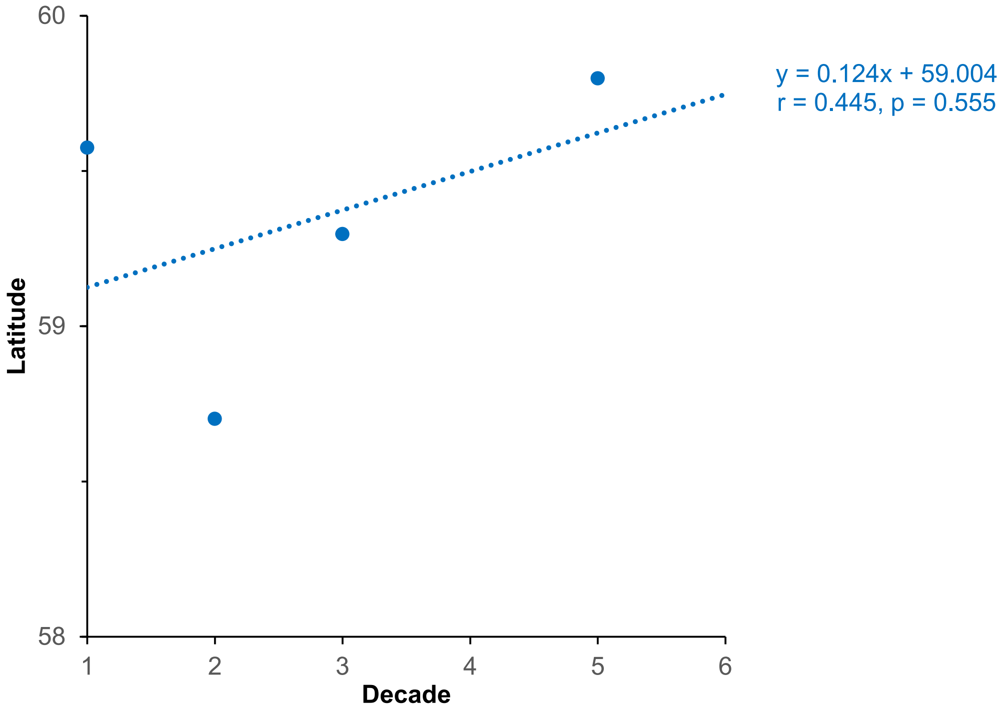

Figure S24. Trends of shifts of the southern boundaries of the sandpaper skate *Bathyraja interrupta* range in the Northwestern Pacific. Designations as in Figure S1.

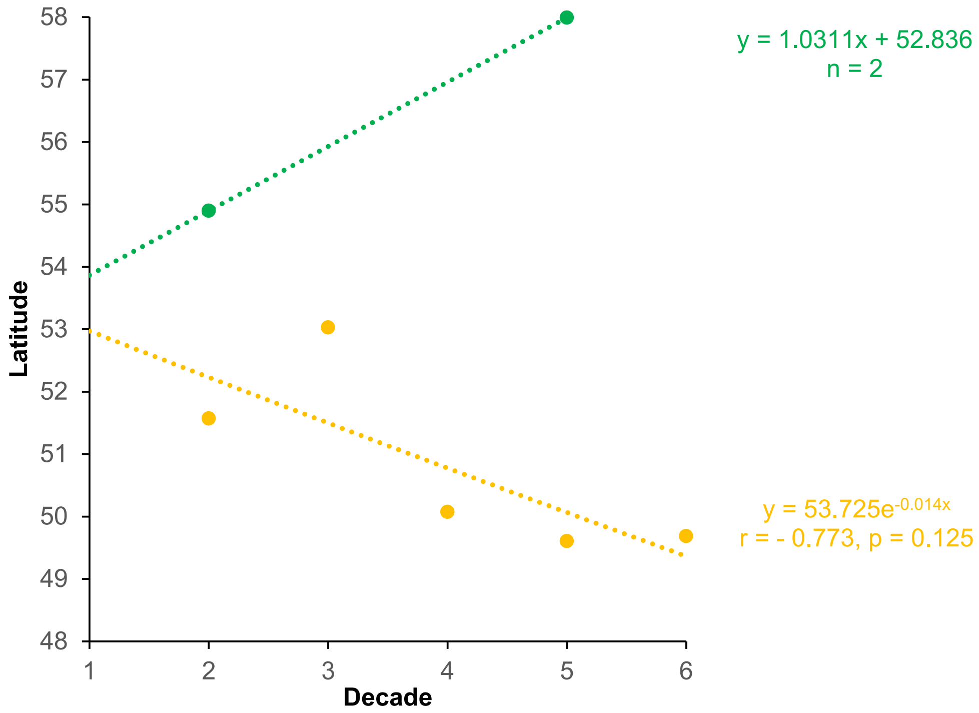

Figure S25. Trends of shifts of the northern boundaries of the challenger's skate *Bathyraja isotrachys* range in the Northwestern Pacific. Designations as in Figure S1.

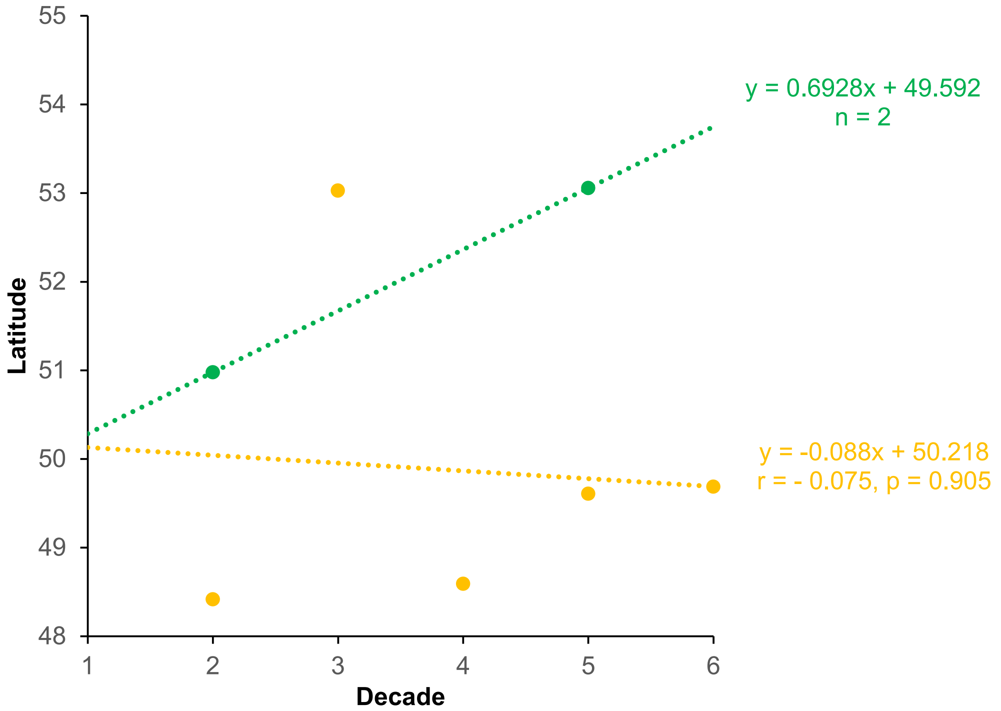

Figure S26. Trends of shifts of the center of the challenger's skate *Bathyraja isotrachys* range in the Northwestern Pacific. Designations as in Figure S1.

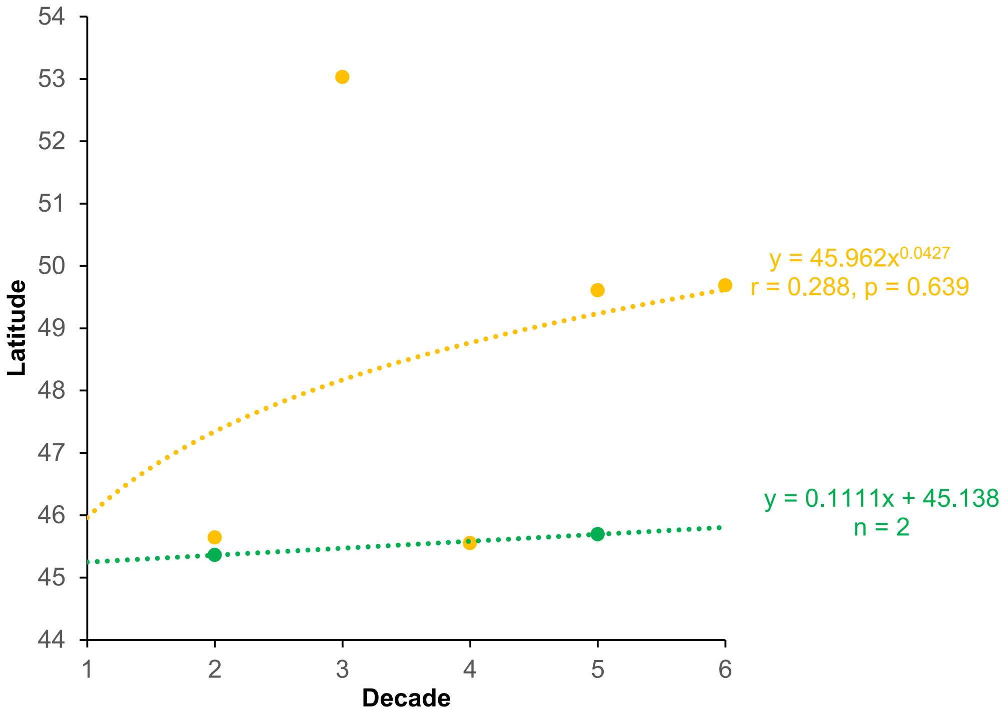

Figure S27. Trends of shifts of the southern boundaries of the challenger's skate *Bathyraja isotrachys* range in the Northwestern Pacific. Designations as in Figure S1.

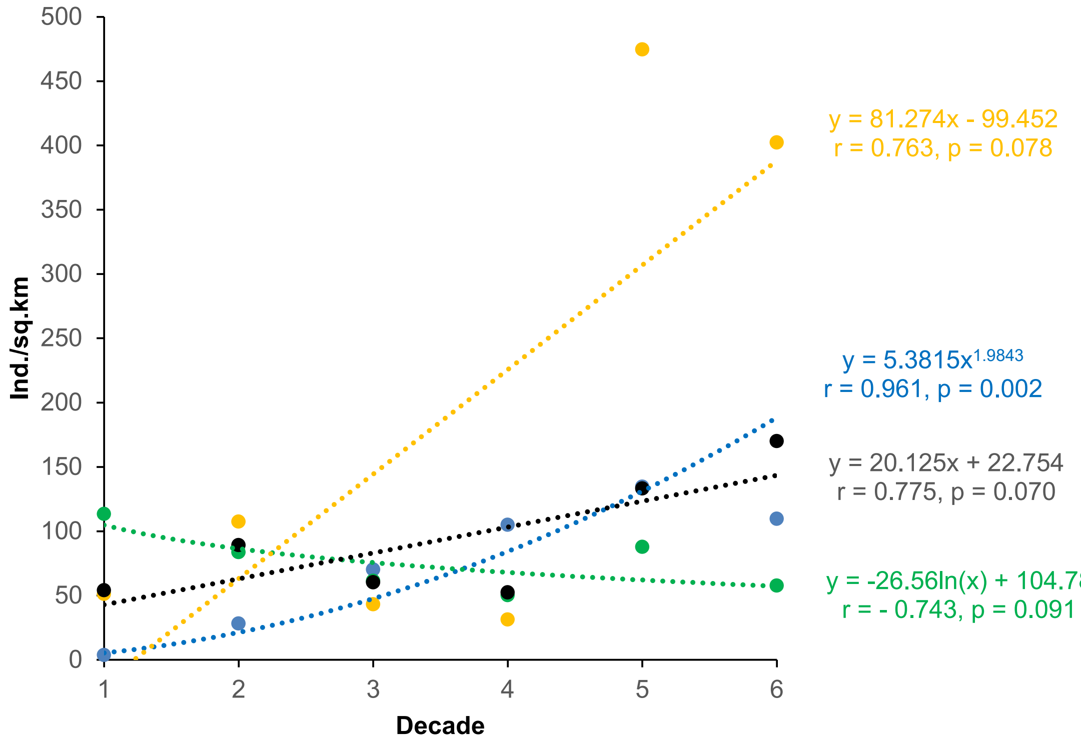

Figure S28. Trends of CPUE changes of the Okhotsk skate *Bathyraja violacea* in the Northwestern Pacific. Here and on the Figs. 29-36 the Bering Sea is in blue, the Sea of Okhotsk is in green, the Sea of Japan is in red, the Pacific Ocean is in yellow, and all areas combined in black. The solid lines with circles indicate the average CPUEs, and the dotted lines indicate the 95% confidence intervals.

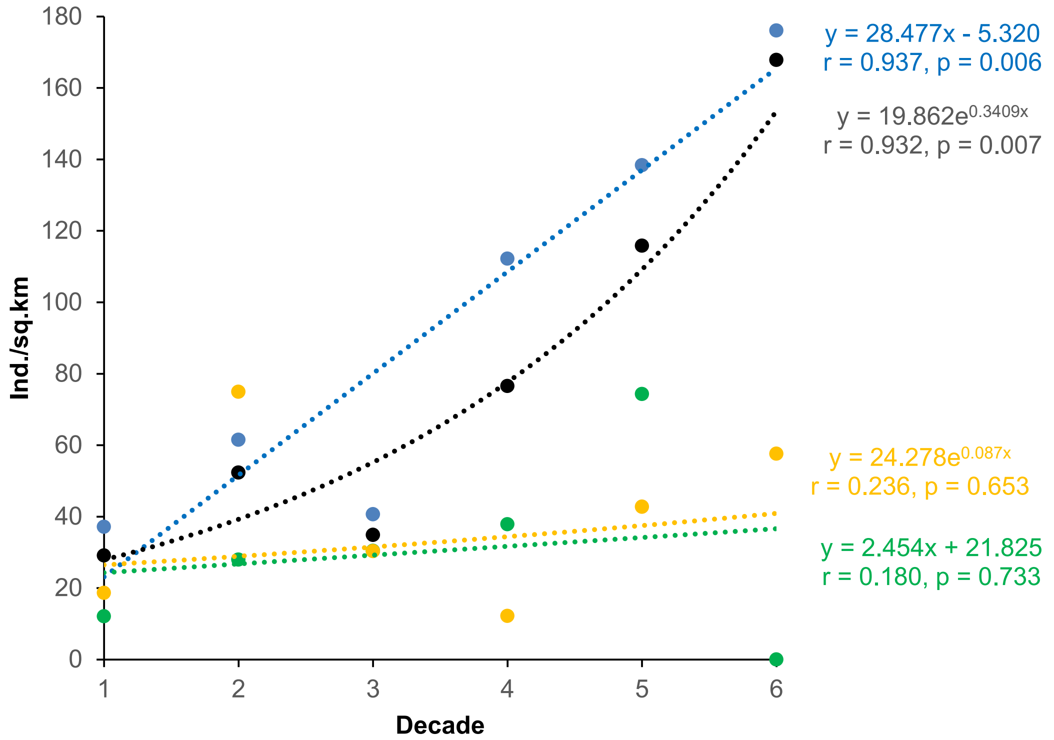

Figure S29. Trends of CPUE changes of the Aleutian skate *Bathyraja aleutica* in the Northwestern Pacific. Designations as in Figure S28.

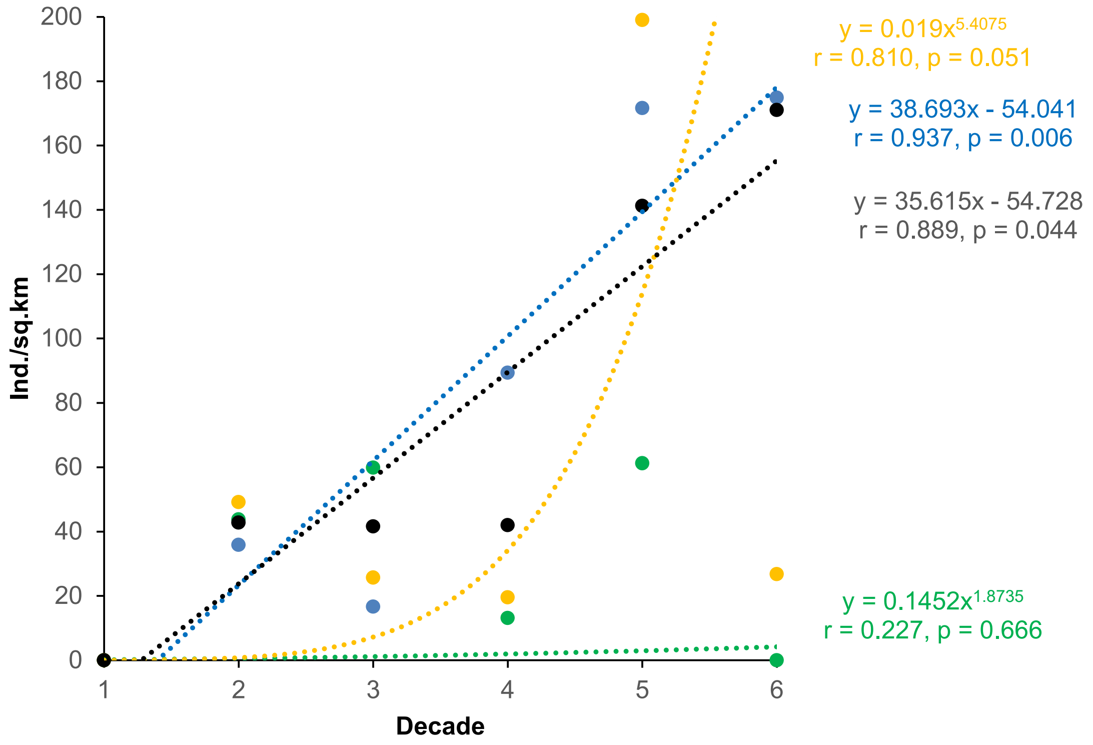

Figure S30. Trends of CPUE changes of the dusky-purple skate *Bathyraja matsubarai* in the Northwestern Pacific. Designations as in Figure S28.

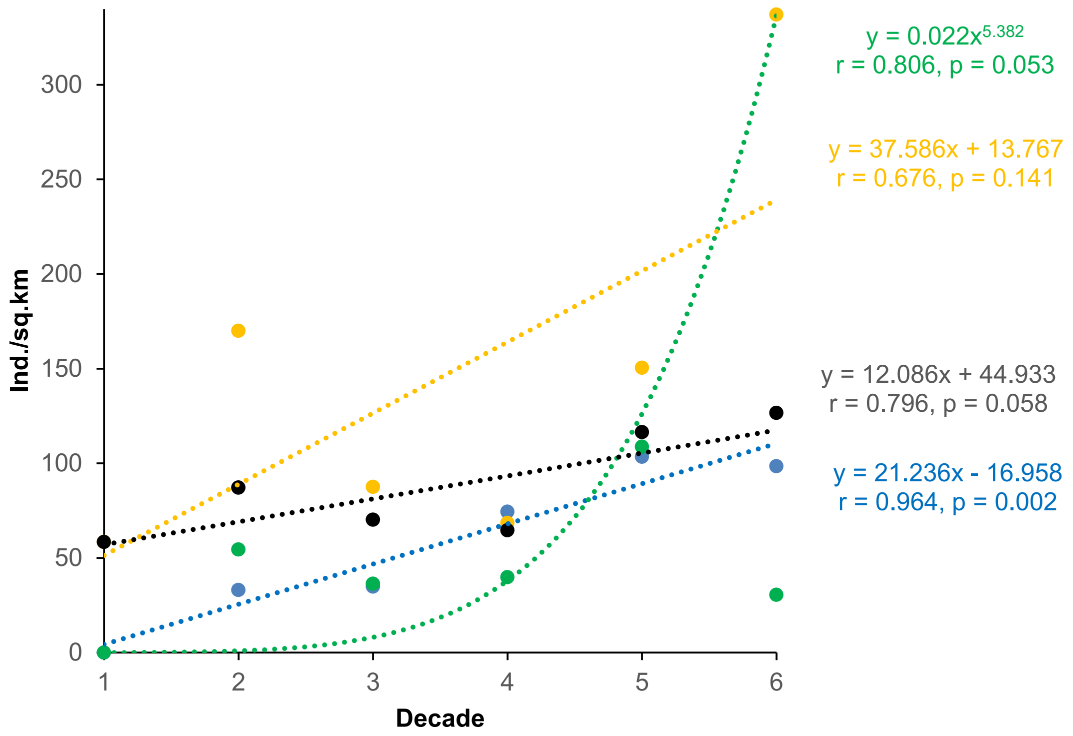

Figure S31. Trends of CPUE changes of the whiteblotched skate *Bathyraja maculata* in the Northwestern Pacific. Designations as in Figure S28.

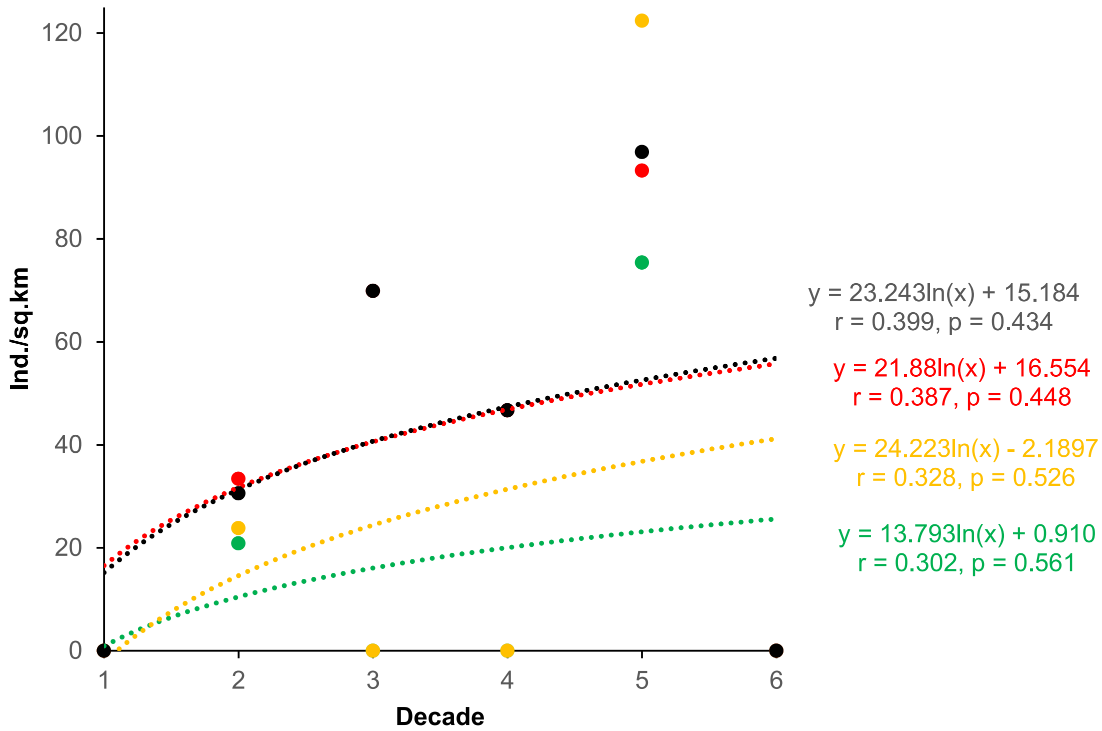

Figure S32. Trends of CPUE changes of the bottom skate *Bathyraja bergi* in the Northwestern Pacific. Designations as in Figure S28.

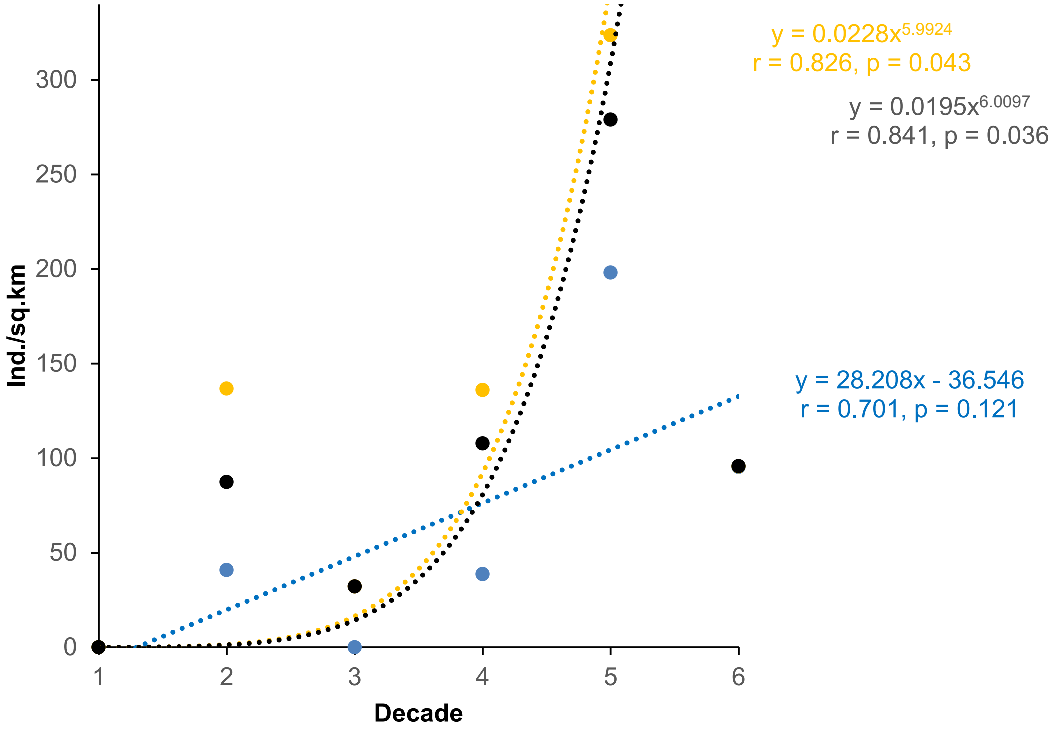

Figure S33. Trends of CPUE changes of the mud skate *Bathyraja taranetzi* in the Northwestern Pacific. Designations as in Figure S28.

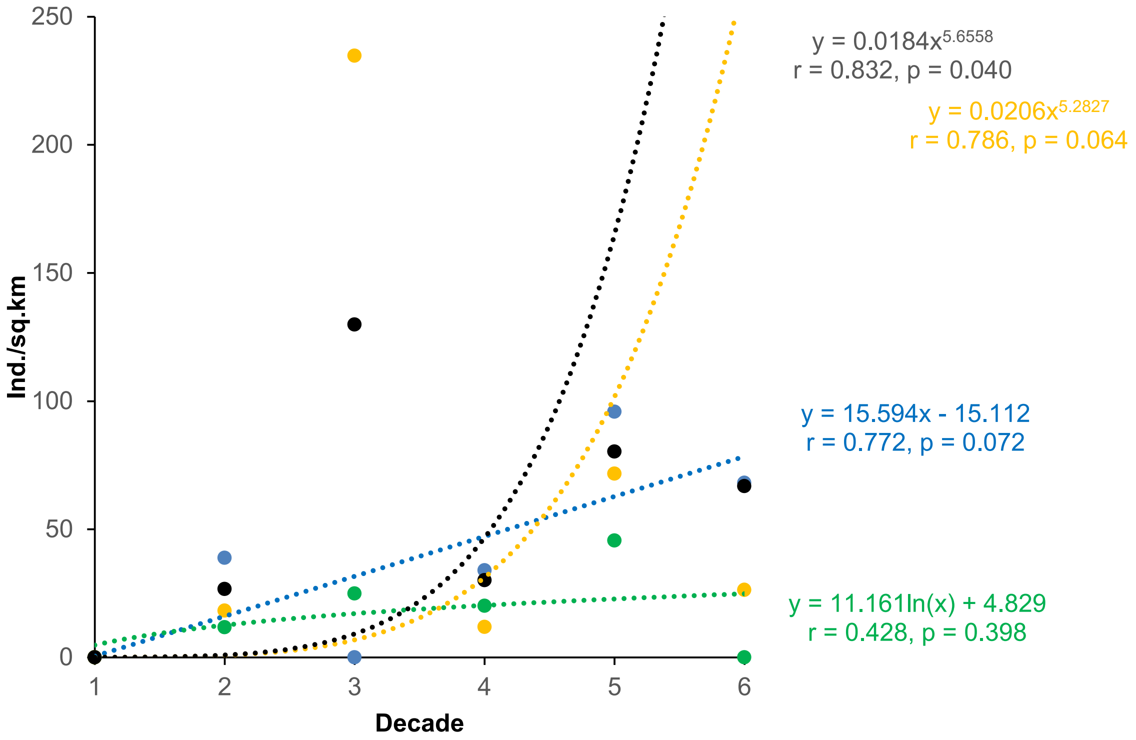

Figure S34. Trends of CPUE changes of the whitebrow skate *Bathyraja minispinosa* in the Northwestern Pacific. Designations as in Figure S28.

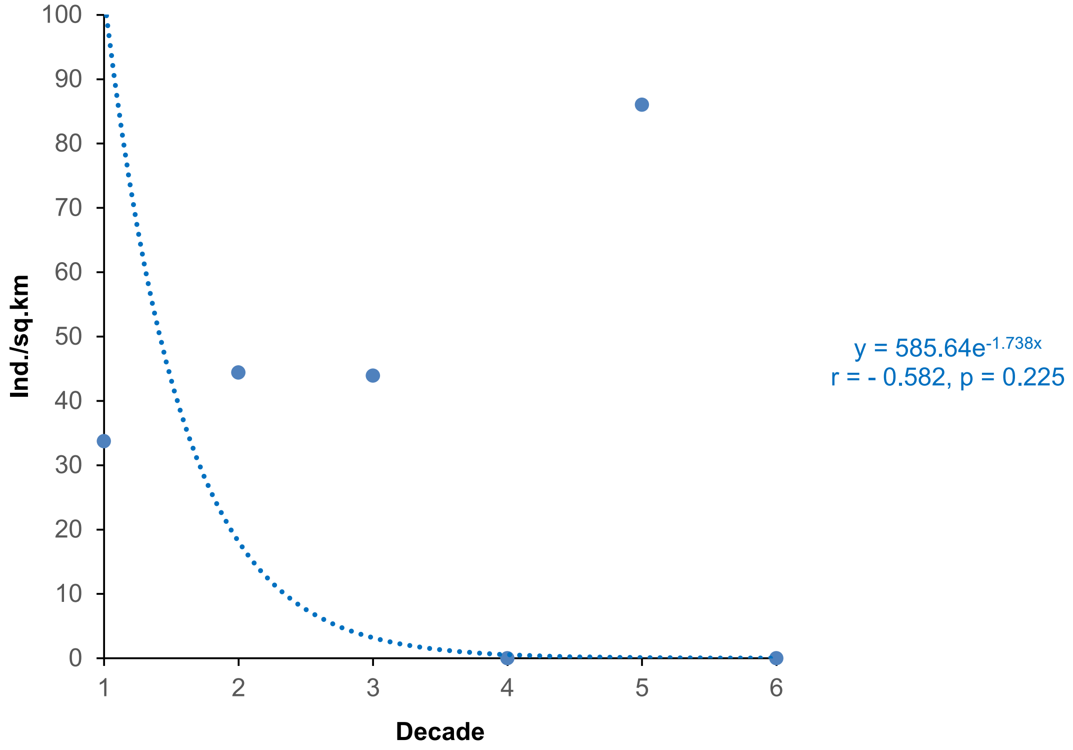

Figure S35. Trends of CPUE changes of the sandpaper skate *Bathyraja interrupta* in the Northwestern Pacific. Designations as in Figure S28.

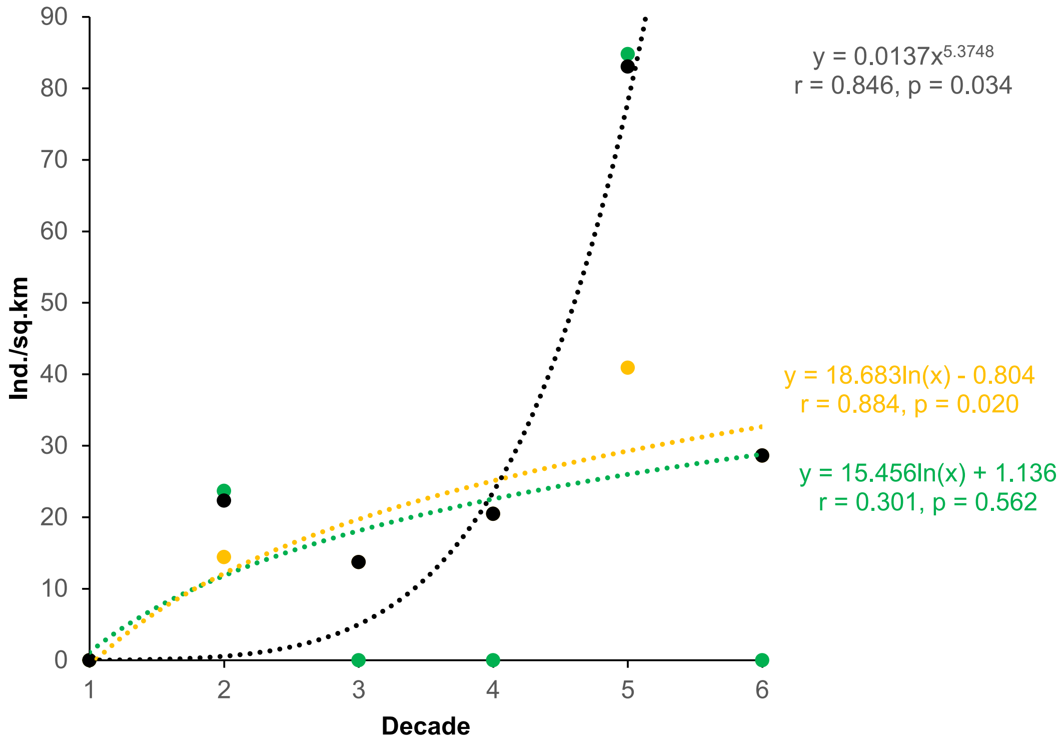

Figure S36. Trends of CPUE changes of the challenger's skate *Bathyraja isotrachys* in the Northwestern Pacific. Designations as in Figure S28.
